# Polyesters and Polyester Nano- and Microcarriers for Drug Delivery

**DOI:** 10.3390/polym16172503

**Published:** 2024-09-03

**Authors:** Stanislaw Slomkowski, Teresa Basinska, Mariusz Gadzinowski, Damian Mickiewicz

**Affiliations:** Division of Functional Polymers and Polymer Materials, Centre of Molecular and Macromolecular Studies, Polish Academy of Sciences, H. Sienkiewicza 112, 90-363 Lodz, Poland; teresa.basinska@cbmm.lodz.pl (T.B.); mariusz.gadzinowski@cbmm.lodz.pl (M.G.); damian.mickiewicz@cbmm.lodz.pl (D.M.)

**Keywords:** poly(β-butyrolactone), polylactide, poly(ε caprolactone), macrolactone, drug carrier, nanoparticle preparation

## Abstract

Many therapies require the transport of therapeutic compounds or substances encapsulated in carriers that reduce or, if possible, eliminate their direct contact with healthy tissue and components of the immune system, which may react to them as something foreign and dangerous to the patient’s body. To date, inorganic nanoparticles, solid lipids, micelles and micellar aggregates, liposomes, polymeric micelles, and other polymer assemblies were tested as drug carriers. Specifically, using polymers creates a variety of options to prepare nanocarriers tailored to the chosen needs. Among polymers, aliphatic polyesters are a particularly important group. The review discusses controlled synthesis of poly(*β*-butyrolactone)s, polylactides, polyglycolide, poly(ε-caprolactone), and copolymers containing polymacrolactone units with double bonds suitable for preparation of functionalized nanoparticles. Discussed are syntheses of aliphatic polymers with controlled molar masses ranging from a few thousand to 10^6^ and, in the case of polyesters with chiral centers in the chains, with controlled microstructure. The review presents also a collection of methods useful for the preparation of the drug-loaded nanocarriers: classical, developed and mastered more recently (e.g., nanoprecipitation), and forgotten but still with great potential (by the direct synthesis of the drug-loaded nanoparticles in the process comprising monomer and drug). The article describes also in-vitro and model in-vivo studies for the brain-targeted drugs based on polyester-containing nanocarriers and presents a brief update on the clinical studies and the polyester nanocarrier formulation approved for application in the clinics in South Korea for the treatment of breast, lung, and ovarian cancers.

## 1. Introduction

There are many reasons for using bioactive substances hidden in carriers. Several classes of bioactive compounds, for example, ifosfamide, cisplatin, or doxorubicin, designed for use as cytostatic or cytotoxic components of anticancer drugs, should be encapsulated in nanocarriers, shielding healthy cells from the drug’s action and thus making it more selective when targeted to cancer cells [1,2,3].

The bioactive compounds should be entrapped in the nanocarriers also when the patient’s organism recognizes them as a “foreign” material that should be destroyed or other way eliminated by the immune system. This is rather common when nucleic acids, proteins, medium oligonucleotides, or oligopeptides are used as drugs [4,5,6].

Nanocarriers have been specifically designed for transport across various barriers in the body, such as the very tight blood-brain [7,8], blood-spinal cord [9], and blood-peripheral nerve barriers [10] or membranes that separate cells from their surroundings [11,12].

Of particular interest are carriers equipped with molecular structures on their surface that bind to receptors specific to cancer cells [13,14,15,16,17].

Importantly, there are so-called “stealth” nanocarriers, i.e., nanocarriers that are not recognized by the patients’ immune system and therefore remain in the blood circulation for a longer time [18,19,20]. To this list should be added carriers that disintegrate, releasing their contents under the influence of external or internal stimuli, such as local temperature change, pH, glucose, glutathione, enzymes, light, ultrasound, and magnetic field [21,22,23,24]. Also of interest are superparamagnetic nanoparticles (SPION), which can be easily “immobilized” in a selected tissue (such as a tumor). Then, when subjected to an alternating electromagnetic field, they increase their temperature and, by hyperthermia, destroy the surrounding cancer cells [25].

After fulfilling their function of transporting bioactive compounds and releasing them at the required location, the carries should be decomposed and eliminated to avoid the undesirable accumulation of their residues. Most often, drug carriers are prepared from synthetic or natural compounds of high molar mass, which, with time, are suitable for hydrolytic degradation. It is worth noting that chemical bonds can be ordered concerning their increased susceptibility to hydrolysis as follows: carbon-carbon bond in aliphatic hydrocarbons < amide < carbonate ≤ ester < anhydride. bonds Aliphatic hydrocarbons and polyamides under normal conditions (human body temperature, aqueous environment) are resistant to hydrolysis and therefore are not suitable for the preparation of drug carriers. Of all other synthetic polymers, aliphatic polyesters are the most promising. They represent the largest and most diverse group, including polymers approved by the US Food and Drug Administration for medical applications.

Susceptibility to hydrolysis is not the only requirement that polymer carriers should meet. They should also be mechanically and thermally stable to ensure convenient processing under various conditions. Moreover, the polymers used for the fabrication of drug carriers should be free of any by-products and traces of unreacted monomers. Scientists are also concerned about the possible distant harmful effects of remaining catalyst and initiator fragments (free or chemically bound) in polymeric materials used to make drug carriers.

According to the Web of Science database, out of thousands of papers published in the past ten years on the polymer drug nanocarriers, 35% were made of aliphatic polyesters. This review focuses on the synthesis and properties of aliphatic polyesters that are used or have the potential to be used in the production of drug carriers. Advances in the development of methods for preparing drug carrier systems that are suitable for encapsulating drugs with various properties are discussed. The review ends with a brief presentation of examples of carriers for targeted drug delivery and conclusions.

## 2. Polyesters for Fabrication of Drug Carriers

**Figure 1:**
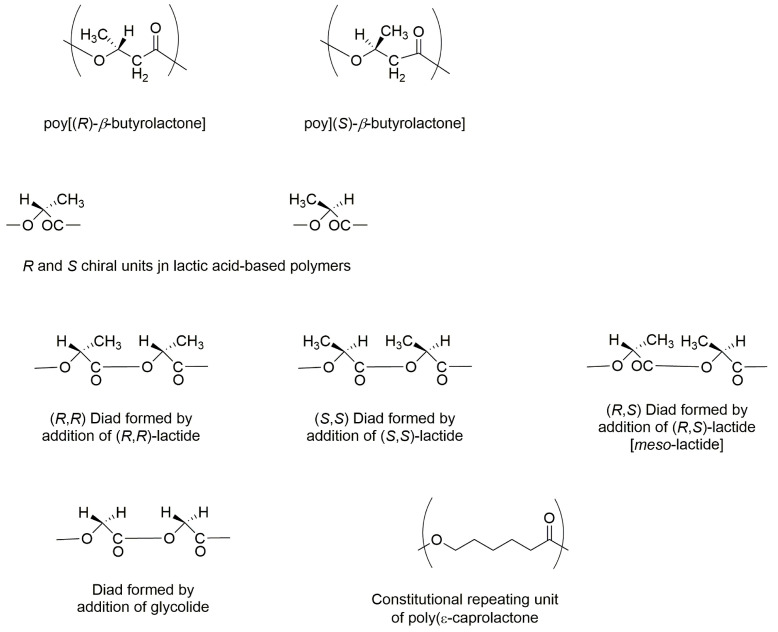
Structures of polymers, which are most often used for the preparation of carriers of drugs and other bioactive compounds.

Structures of polyester blocks in homo- and copolymers traditionally used for the preparation of nano- and microparticulate carriers of bioactive substances are shown in Figure 1.

It is worth noting that each constitutional repeating unit (CRU) of poly(*β*-butyrolactone) and polylactide contains a chiral carbon atom (with *R* or *S* configuration), which broadens possible variation of chain microstructure affecting polymer properties such as the ability to crystallize and degrade as well as their thermo-mechanical behavior and, as a result, a field of their applicability.

### 2.1. Synthesis of Poly(β-Butyrolactone) (Other Name Poly(Hydroxybutyrate))

There are three major routes for the synthesis of aliphatic polyesters: ring-opening polymerization of cyclic esters (lactones and lactides), polycondensation of hydroxycarboxylic acids, and bio-related methods. Poly(*β*-butyrolactone) (often referred to as poly(hydroxybutyrate)—PHB) is produced by over twenty various strains of microbes [26,27,28,29,30]. Later, in the review, the acronym PHB will be used for bacterial and plant-produced polymers, whereas PBL (poly(*β*-butyrolactone)) for polymers produced by invitro methods.

The polymer synthesized inside of bacteria aggregates into granules, accounting for up to 90% (wt) of cell dry weight [31]. Molar masses of bacterial PHB are usually in the range of 9 × 10^3^ to 2.4 × 10^6^ g/mol [32]. Polymers with much lower molar masses, often oligomers, are usually needed to produce drug delivery carriers. Therefore, it is worth noting that high-molar-mass polyesters, which like PHB belong to the polyhydroxyalkanoate family, can be enzymatically (e.g., by *Pseudomonas putida (oleovorans)*) hydrolyzed or catalytically (using ethylene glycol/dibutyltin dilaurate) transesterified to low-molar-mass products [33,34].

PHB granules were also produced by transgenic plants created by the introduction of bacterial genes. For example, by alfalfa modified by introduction of genes (*phb*A, *phb*B, *phb*C) from *Ralstonia eutropha* [35], sugar beet clone 93161p with introduced plasmid pRi15834 from *Agrobacterium tumefaciens* [36], flax with cDNA encoding the *β*-ketothiolase or phbA, phbB, phbC genes [37], oil palm by introduction of phbA, phbB, phbC genes [38] and by transgenic sugarcane (*Saccharum* sp. cv Q117) and switchgrass (*Panicum virgatum* L.) plants [39]. However, PHB content in harvested biomass was usually below 4% dry weight content of harvested biomass. Only quite recently some transgenic *Camelina sativa* plants (known also as false flax) with seeds containing up to 15.2% (*wt*/*wt*) of PHB were obtained [40]. However, this value is still very much lower than the aforementioned 90% (wt) produced by bacteria.

It is worth mentioning that the vast majority of bacteria and plants produce PHB only with the main chain containing only *R* stereocenters and therefore pure isotactic. Synthetic methods make it possible to obtain *R* stereoisomers and a variety of *R/S* polymers with different microstructures.

The polymerization of racemic *β*-butyrolactone carried in bulk and initiated with Et_2_Zn-H_2_O was found to lead to a product that consists of two fractions, the one soluble in chloroform and the other insoluble in this solvent [41]. The soluble fraction turned out to be non-crystalline and optically inactive. This means that the said PBL is racemic and atactic. The chloroform-insoluble fraction was also optically inactive, but its X-ray spectra were essentially identical to those of the bacterial PHB, which contains only *R* stereocenters. The conclusion was that this fraction consists only of isotactic chains (or chains with very long isotactic blocks) with about the same content of *R* and *S* stereo-centers.

Shelton et al. reported on the synthesis of *β*-butyrolactone enriched in *R* isomer (about 73% content) and showed that in polymerization initiated with the Et_3_Al-H_2_O system, this monomer can be converted to an optically active polymer similar to natural (i.e., bacterial) PHB [42].

Comprehensive studies of the polymerization of racemic *β*-butyrolactone and other *β*-alkyl-*β*-propiolactones initiated with Et_3_Al-H_2_O and Et_2_Zn-H_2_O initiators with various proportions of (metal alkyl)-water components have greatly improved the understanding of the mechanism of polymerization but have not led to the required level of stereospecificity of this process [43,44].

Le Borgne and Spassky reported the results of their studies on the stereoelective polymerization of racemic *β*-butyrolactone initiated with products of reactions of ZnEt_2_, AlEt_3_, and CdMe_2_ with the *R* enantiomer of 3,3 dimethyl-l,2 butanediol (*R*(-)-DMBD) [45]. It was found that all polymerizations were stereoelective but differed concerning the efficiency and character of stereoelectivity. The most efficient was the ZnEt_2_/*R*(-)-DMBD system leading to homosteric polymerization (polymerization with asymmetric carbon atoms in the initiator and in the preferentially incorporated lactone isomer being the same) and with the stereoelectivity ratio (a measure of preferential consumption of a given enantiomer) *r*_R_ = 1.7. Polymerization initiated with the AlEt_3_/*R*(-)-DMBD system was also homochiral but its stereoelectivity was lower (*r*_R_ = 1.1). It should be noted that the CdMe_2_/*R*(-)-DMBD system initiates the antisteric polymerization. (Configurations of chiral carbon atoms in initiator and preferentially incorporated monomer were opposite); however, its stereoelectivity was very low (*r*_R_ = 1.01).

**Scheme 1:**
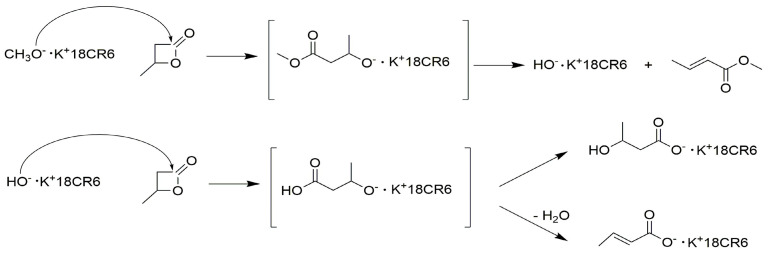
Polymerization of *β*-butyrolactone—mechanism of initiation with CH_3_O^–^K^+^ˑ18CR6.

**Scheme 2:**
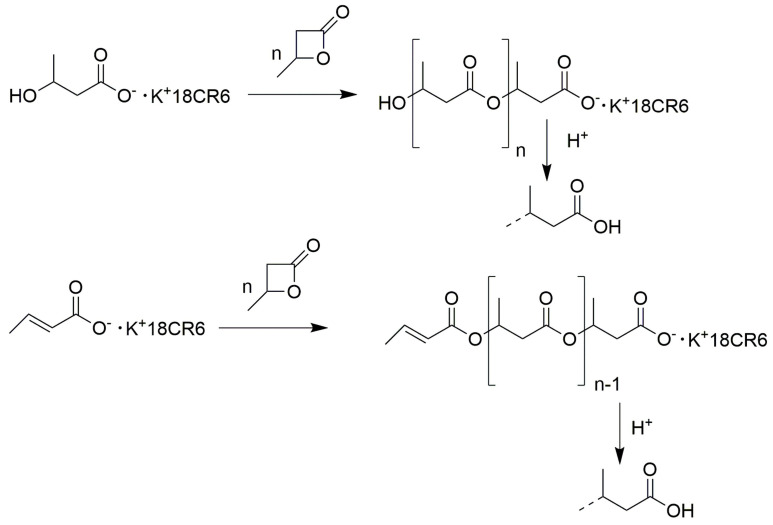
Polymerization of *β*-butyrolactone—propagation.

It has been noticed that alkoxides and carboxylic acid salts with sodium or potassium cations do not initiate anionic polymerization of *β*-butyrolactone [46]. However, complexing the aforementioned cations by crown ethers with the proper size of cavity is known to convert tight ion pairs to the loose ones, facilitating ion pair dissociation to free ions, making these compounds efficient initiators of the *β*-butyrolactone polymerization. The polymerization initiated with CH_3_O^−^K^+^ˑ18crown6 (CH_3_O^−^K^+^ˑ18CR6) is a good example. In this case, although the initiation is relatively complicated (see Scheme 1), the propagation consists of the simple addition of monomer molecules to the carboxylate active centers, proceeding with cleavage of the alkyl-oxygen bond [47]. The propagation is shown in Scheme 2. The resulting macromolecules have HO- or crotonate ester end-groups at one and carboxylic acid groups at the other end.

Several attempts were undertaken to obtain synthetic PBL [poly(*β*-butyrolactone)] with exclusively (or mainly) ^®^ stereo-centers, i.e., the polymers resembling natural PHB. Very successful in this research were Lenz et al., who used Et_3_Al-H_2_O and Et_2_Zn-H_2_O as initiators polymerized (*S*)-*β*-butyrolactone. In these processes the propagation occurred with alkyl-oxygen bond scission, with inversion of configuration, yielding eventually ^®^-PBL [48]. In the same paper, authors noted that in polymerization of (*S*)-*β*-butyrolactone catalyzed with ethylaluminoxane, the monomer is added via the acyl-oxygen bond scission route with retention of configuration [(*S*)-PBL was produced].

It should be mentioned that although polymerizations of (*S*)-*β*-butyrolactone initiated with alkali metal alkoxides yield polymers almost exclusively with *R* stereocenters, the products still may differ from the natural ones. Whereas the natural PHB contains only HO-… …-COOH groups, the synthetic one may also contain a fraction of macromolecules with crotonate ester end-groups (CH_s_CH=CHCOO-… …-COOH; see Scheme 2). However, it was found that polymerization of (*S*)-*β*-butyrolactone initiated with ^®^-3-hydroxybutyric acid sodiumˑ (crown ether) salt gave polymer containing only end-groups, which are present in natural PHB [21].

Bacterial and plant polyhydroxybutyrates with pure isotactic structures have good mechanical properties, enabling their application as degradable replacements of conventional commodity thermoplastics such as polyethylene and polypropylene and as specialty polymers for usage in medicine [49]. However, these polymers also have some undesirable properties. One of them is brittleness [50,51]. Moreover, their melting temperature (about 180 °C) is very close to the temperature at which thermal degradation becomes important, which makes the processing from melt difficult [32,50,52]. The problem was usually solved by using additives, decreasing polymer melting temperature and/or increasing temperature, at which polymer begins to degrade. However, in the case of polymers for medical applications, such an approach would require the selection of not only biocompatible polymers but biocompatible additives as well. Thus, an interesting alternative was the development of the synthesis of preferentially syndiotactic poly(*β*-butyrolactone) with low melting temperatures (usually below 120 °C) by using racemic (*R*, *S*)-*β*-butyrolactone as monomer and distannoxane derivatives as catalysts [53].

**Figure 2:**
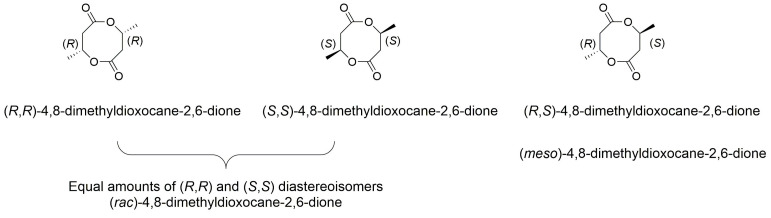
Structures of the cyclic dimers of *β*-butyrolactone.

Recently, important progress in the synthesis of poly(*β*-butyrolactone)s with various stereo configurations was achieved by Chen et al. [54]. These studies included the synthesis of a mixture of cyclic dimers of *β*-butyrolactone (4,8-dimethyldioxocane-2,6-dione) with structures shown in Figure 2.

From the mixture, they isolated by crystallization pure racemate [(*R*,*R*)-4,8-dimethyldioxocane-2,6-dione + (*S*,*S*)-4,8-dimethyldioxocane-2,6-dione] and by column chromatography and crystallization pure (*R*,*S*)-4,8-dimethyldioxocane-2,6-dione *meso*-diastereoisomer. By using complex initiators inducing stereoselective polymerization of (*rac*)-4,8-dimethyldioxocane-2,6-dione, they obtained a racemic mixture of crystalline isotactic poly[^®^-*β*-butyrolactone] and poly[(*S*)-*β*-butyrolactone]. Polymerization of (*meso*)-4,8-dimethyldioxocane-2,6-dione yielded an amorphous pure syndiotactic polymer. Diastereoselective copolymerization of (*rac*)-4,8-dimethyldioxocane-2,6-dione with various amounts of added at start (*meso*)-4,8-dimethyldioxocane-2,6-dione enabled synthesis of stereosequenced poly(*β*-butyrolactones) with modified mechanical and thermal properties.

### 2.2. Polylactides

Aliphatic polyesters derived from lactic acid enantiomers, or their cyclic dimers are an important class of polymers used in medicine. The chains of these polymers contain the same *R* and *S* stereorepeating units, regardless of whether they were obtained from lactic acids or lactides. In both cases, the term polylactides is commonly used.

Oligolactides were first obtained by Gay-Lussac and Pelouze in the 19th century as by-products formed during the drying of lactic acid by distillation [55]. However, studies directed toward determining the mechanism of polymerization of lactones and lactides were not conducted until about a century later by Carothers et al. [56]. Unfortunately, the polyesters obtained in these studies had low molar masses and were susceptible to hydrolysis. Both of these properties were considered highly undesirable, and ultimately polylactides did not attract much attention at the time. Even later, during two decades from 1945–1964, only four papers mentioning polylactides have been published. The great interest in polylactides started in the last two decades of the 20th century, during which, according to the Web of Science database, the number of publications on polylactides reached about 3500. The field is still very hot, with the average number of papers published per week approaching 80.

Among the approximately forty thousand journal publications and book chapters on PLA that have appeared in the last twenty years, there have been several very comprehensive reviews [57,58,59,60,61]. It is also worth mentioning the recent IUPAC recommendations for nomenclature and terminology for linear lactic acid-based polymers [38].

In this review, we do not intend to discuss in detail the existing knowledge on the synthesis and properties of PLA, but we briefly summarize the most important information relevant to the application of polylactides as matrices for drug carriers.

**Figure 3:**
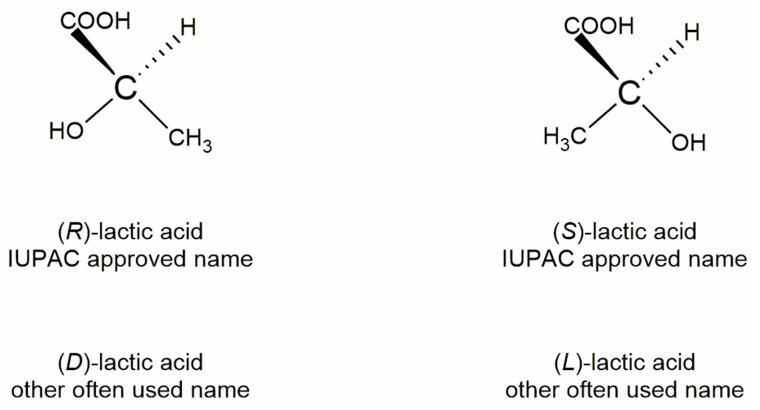
Structures lactic acid stereoisomers. Names according to Ref. [62].

The main raw material for the synthesis of polylactides is lactic acid. There are two stereoisomers of this compound (see Figure 3).

Lactic acid is produced on a mass scale by biotechnological processes (lactic acid fermentation). The following bacteria strains were used for the synthesis of ^®^-lactic acid: *Lactobacillus delbrueckii* LD0025, *Lactobacillus delbrueckii bulgaricus* (LB12), *Lactobacillus coryniformis subsp. torquens* (DSM20004), *Sporolactobacillus inulinus* (NBRC13595), *Lactobacillus coryniformis* (NCDC369), Sporolactobacillus inulinus SI0073, metabolically engineered *Lactobacillus plantarum*, and *Lactobacillus coryniformis subsp. torquens* (DSM20004) [63,64,65,66,67].

There are also strains of bacteria that produce (*S*)-lactic acid: *Alkaliphilic Bacillus* sp. WL-S20, Bacillus coaggulaans, Bacillus subtilis MUR1, Candida sonorensis, Lactococcus lactis LL0018, *Lactobacillus casei* sp., and recombinant *Escherichia coli* [63,68,69].

Recent advances in microbial lactic acid synthesis, purification process, and purity of final products are significant. Namely, the efficiency of sugar conversion to *R*-lactic acid approached 90%, and the optical purity of the resulting product was 99.9% [67].

However, it is also worth mentioning the chemical synthesis of racemic lactic acid from ethylene in a process consisting of three steps: oxidation of ethylene to acetaldehyde in a process catalyzed with PdCl_2_, catalytic conversion of acetaldehyde to lactonitrile in reaction with hydrogen cyanide, and hydrolysis of lactonitrile using sulfuric acid as a catalyst [70].

Seemingly the simplest way to synthesize polylactides should be polycondensation of lactic acids. However, this process faces a significant difficulty. In the polycondensation of hydroxyacids, every additional step is accompanied by the formation of a water molecule. Since polycondensation of hydroxyacids is reversible, without the removal of water, the process would reach equilibrium, at which only short polymer chains would be present in the system. However, when the polycondensations were carried out in the presence of catalysts in a high boiling solvent (diphenyl ether) at 130 °C with the removal of water by azeotropic distillation and turning back the dried solvent, polylactides were formed. If after 24 h of the above process, the molar mass of the polylactides was still low (*M*_w_ about 5000 g/mol), the polycondensation was continued for an additional 16 h at 160 °C, yielding polylactides with *M*_w_ ranging usually from 10^4^ to 10^5^ g/mol, depending on the catalyst [71]. Many compounds have been tested as catalysts for this solution polycondensation, including protonic acids (H_3_PO_4_, H_2_SO_4_, CH_3_SO_3_H, p-toluenesulfonic acid, Nafion-H^®^ ), metals/metalloids (Al, Mg, Sn, Ti, Zn), metal/metalloid derivatives such as oxides (GeO_2_, Sb_2_O_3_, SnO, SnO_2_, TiO_2_, ZnO, ZrO_2_); chlorides (SnCl_2_, SnCl_4_, ZnCl_2_); salts of metals/metalloins and organic acids such as acetic acid (AcO), lactic acid (LA), oleic acid (OA) e.g., Co(AcO)_2_, Cu(OA)_2_, Fe_2_(LA)_3_, Mn(AcO)_2_, Ni(AcO)_2_, Ti(acac)_2_, Y(OA)_3_, Zn(LA)_2_; and alkoxides Al(*i*-PrO)*_3_*, Ti(BuO)_4_ and also (Bu)_2_SnO [71]. The polylactides with the highest molar masses (*M*_w_ from 230,000 to 240,000 g/mol) were obtained using Sn, SnO, and SnCl_2_ catalysts.

It should be mentioned that in 1995 the high-temperature solution condensation process was commercialized by Mitsui Chemicals Co. (Tokyo, Japan). However, production was suspended a few years later.

In 2001, Kimura et al. reported on the development of a melt/solid polycondensation of the (*S*)-lactic acid yielding (*S*)-PLA with *M*_w_ reaching 600,000 g/mol (determined by GPC using polystyrene standards for calibration) [72,73]. The process begins with the classical thermal dehydration of monomers with the formation of the oligomers with a degree of polymerization of about 8. To this mixture were added SnCl_2_ˑ2H_2_O) and *p*-toluenesulfonic acid. Then, the temperature was increased to 180 °C, and the polycondensation was carried out in the molten state at pressure that decreased gradually to 10 Torr with stirring for 5 h. After this time, the *M*_w_ of the polymer was 13,000 g/mol, and the polymerization mixture was cooled to room temperature. The prepolymer was then heated to 105 °C and kept at this temperature for 1 or 2 h. At this stage, the prepolymer crystalized. During crystallization, the polymer chains segregate. The amorphous phase should be more abundant in shorter chains and the crystalline phase in longer chains. As a result, the local concentration of the hydroxyl and carboxyl end-groups should be high in the amorphous phase. Moreover, the end-groups of the crystalline phase chains should be located preferentially in the interfacial areas. In this way, the aforementioned segregation of chains should facilitate polycondensation. After the crystallization step, the polycondensation of the prepolymer was carried out in the solid state at 150 °C for up to 30 h. The highest molar mass (600,000 g/mol) was observed for a crystallization time lasting 2 h and polycondensation lasting 20 h. After a longer (30 h) time, some degradation was observed, and *M*_w_ decreased to about 200,000 g/mol. However, despite evident advantages, this process is not used for the industrial synthesis of (*S*)-polylactide [73].

Currently, the most advanced technology for the synthesis of PLAs is based on ring-opening polymerization of lactides. This process involves lactic acid oligo condensation (initiated, for example, by ZnO or Sn(Oct)_2_ (tin 2-ethylhexanoate)), oligo condensation of lactic acid carried out at 140 °C, catalytic (using ZnO as a catalyst) decomposition of linear PLA oligomers to the cyclic dimers of lactic acids (i.e., lactides), their thorough purification, and their subsequent use as monomers in ring-opening polymerization [61,74,75].

The technology involving the ring-opening polymerization of lactides is the most attractive because it is less demanding and relatively simple. Both polycondensation and ring-opening polymerization are preceded by oligocondensation of lactic acid. However, it is worth noting that oligocondensation does not require a dry monomer. Very good results have been obtained using an aqueous solution with a lactic acid content of only about 40% [50]. In the polycondensation process, the next step is a tedious and energy-demanding, much more advanced dehydration. In the ring-opening process, the second step involves the depolymerization of oligopeptides into lactides, which are easily purified and dried by distillation, sublimation, and crystallization from ethyl acetate and subsequently from ethyl methyl ketone and washing with ice-cold dry ethyl ether [74,75]. The lactides can be distilled and sublimed at temperatures slightly below the thermal decomposition becomes significant, i.e., about 200 °C [76]. The distillation is carried out at a pressure of 0.1–15 Torr and a temperature ranging from 180 to 215 °C, while sublimation is carried out at a pressure of 0.1 Torr and temperatures from 60 to 100 °C [75].

**Figure 4:**
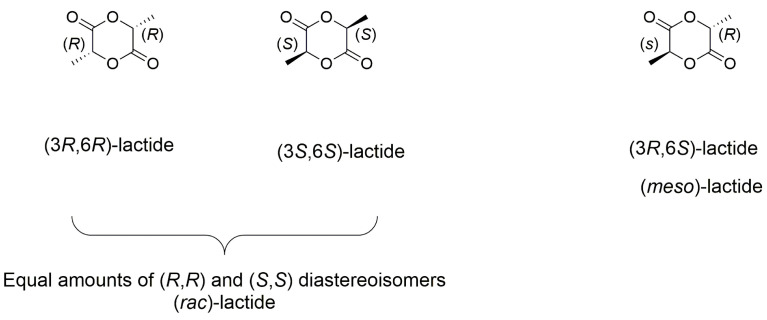
Structures of lactide stereoisomers.

Syntheses of lactides from pure *R* and pure *S* lactic acid stereoisomers yield (*R*,*R*)-lactide and (*S*,*S*)-lactide, respectively. However, similar syntheses from mixtures of *R* and *S* lactic acids in addition to a mixture of (*R*,*R*)-lactide and (*S*,*S*)-lactide diastereoisomers also yield meso-lactide ((*R*,*S*)-lactide). Structures of the aforementioned diastereoisomers are shown in Figure 4. Their proportions depend on the stereoenantiomeric composition of the initial reaction mixture.

Several classes of compounds were positively verified as initiators and initiators/catalysts. They include: for anionic and coordination ring-opening polymerization metal alkoxides, inorganic salts (salts of organic acids)/alcohol systems, metal oxides, so-called organic superbases, and enzymes; for cationic polymerization protic acids. A large number of metal and metalloid alkoxide initiators was discussed in a review by Slomkowski, Penczek, and Duda [60]. In addition to aluminum tris-isopropoxide (Al(O-*i*-Pr)_3_) and tin di-butoxide (Sn(OBu)_2_) to this group belong also many other alkoxides of lithium, sodium, potassium, calcium, magnesium, scandium, scandium, yttrium, lanthanum, titanium hafnium, and zirconium. A characteristic feature of metal alkoxides (both initiators and propagating species) is their tendency to form aggregates that differ in reactivity. For example, Al(O-*i*-Pr)_3_ may exist as a mixture of reactive trimer and much less reactive tetramer [77]. Thus, for controlled polymerization, aluminum tris-isopropoxide with a known proportion of trimer and tetramer should be used. Methods have been developed to obtain both trimer and tetramer as almost pure individual compounds [78]. However, it is also worth mentioning that aggregation is often reduced by preparing alkoxides complexed by compounds with bulky organic groups [60].

**Figure 5:**
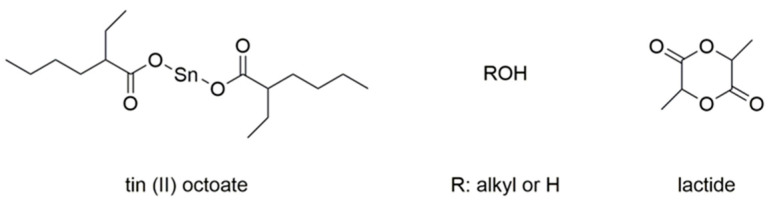
Initial composition of the polymerization mixture.

**Scheme 3:**
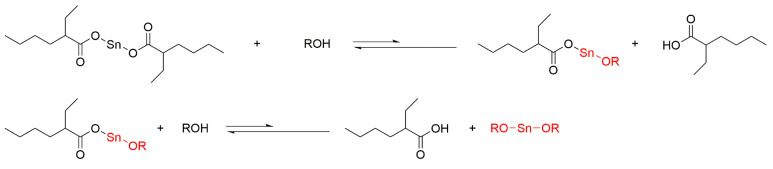
Formation of initiators (red marked are groups participating in initiation).

**Scheme 4:**
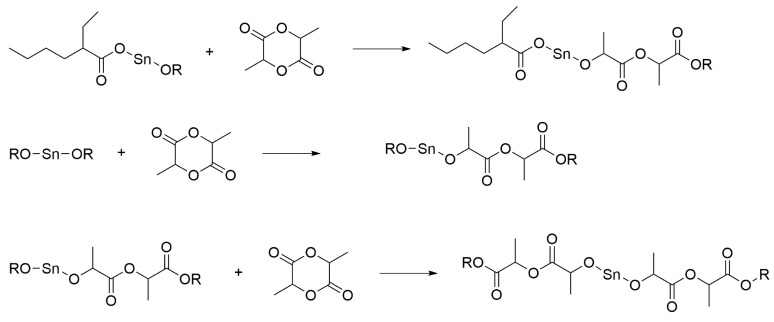
Initiation of lactide polymerization by an initiator produced in situ from the Sn(Oct)_2_ˑROH.

**Scheme 5:**
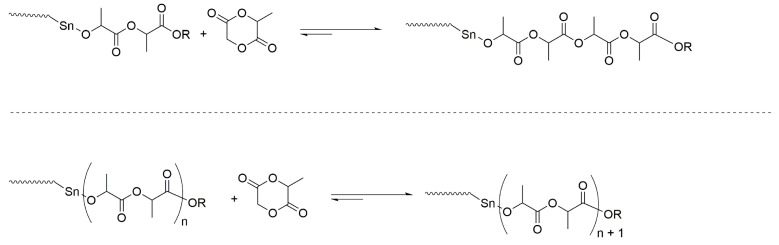
Propagation in lactide polymerization by an initiator produced in situ from the Sn(Oct)_2_ˑ.ROH.

Tin, zinc, calcium, and magnesium salts of 2-ethylhexanoic acid belong to an important group of compounds used to initiate polymerization of lactides [60,79,80]. The most common is the polymerization initiated by tin (II) bis(2-ethylhexanoate) (often called tin (II) octanoate, abbreviated Sn(Oct)_2_). Several, sometimes contradictory, models of the polymerization mechanism based on the use of the said compound have been proposed. Finally, a complete mechanism consistent with all experimental observations was established by Penczek and Duda [81,82]. They noted that for initiation and propagation, tin (II) octanoate alone is not sufficient, but the polymerization mixture must also contain a certain, usually small, amount of alcohol (ROH) or water. Moreover, it has been established that neither tin (II) octanoate nor alcohol alone can initiate the polymerization of lactides. However, the reaction of these compounds produces in situ the true initiating species, tin (II) mono- and di-alkoxides. These compounds (-Sn-OR and RO-Sn-OR) react with the lactide to form the propagating species responsible for chain growth. All reactions involved in the polymerization process are reversible. The rate of polymerization is proportional to the product of the actual lactide concentration and concentration of the tin alkoxide moieties. The concentration of polymer chains is equal to the initial concentration of alcohol molecules. The whole process involving also chain transfer is shown in Figure 5 and Scheme 3, Scheme 4 and Scheme 5.

It has been established that propagation in the polymerization of (*S*,*S*)-lactide is a reversible reaction, and at equilibrium, a certain amount of monomer remains in the system [83]. For example, for polymerization carried out in 1,4-dioxane at temperatures ranging from 80 °C to 135 °C, the equilibrium monomer concentration increases from 0.05 to 0.15 mol/L (i.e., from 5 to 15% of the initial lactide concentration). Such a high content of unreacted monomer is usually unacceptable for polymers used for medical purposes because the presence of the residual lactide has the effect of causing uncontrolled degradation of the polymer and may also affect its interactions with biological systems. Two polymerization methods were developed to obtain poly[(*S*,*S*)-lactide] free of traces of unreacted monomer.

The first method consists of the polymerization initiated using Sn(Oct)_2_ and carried out under two temperature regimes [84]. The polymerization starts at 140 °C in molten monomer. With monomer conversion, the polymerizing mixture solidifies into a supercooled amorphous substance, which, when remained even up to 10 h under the aforementioned conditions, contained 5.6% of unreacted monomer. However, when after the first hour of polymerization at 140 °C the temperature is lowered to 120 °C the crystalline phase is formed and separates from the amorphous one. Finally, after 9 h, the monomer conversion approaches 100%. This occurs because the whole amount of unreacted monomer remains in the amorphous phase and its local concentration exceeds the equilibrium concentration close to the complete monomer conversion. It is even more convenient to perform the polymerization at a higher temperature, i.e., the first step at 170 °C and the second one at 140 °C, because at higher temperatures the [(S,S)-lactide] polymerization proceeds faster.

The second method is based also on a two-step process. In the first step, a simple homopolymerization of (S,S)-lactide is carried out until the monomer concentration approaches the equilibrium concentration. At this stage, the required amount of a second monomer, ε-caprolactone, is added, starting the copolymerization of (*S*,*S*)-lactide and ε-caprolactone [60]. To limit transesterification, the polymerization should be initiated with an initiator that selectively favors propagation over transesterification, e.g., with the reaction product of the trimer of Al[OCH(CH_3_)_2_]_3_ with the (S)-(+)-2,2′-[1,1′-binaphthyl-2,2′-diylbis(nitrylomethilidyne)]diphenol [(S)-SB(OH)2]. It has been shown that when the initial concentration of added ε-caprolactone sufficiently exceeds the equilibrium concentration of lactide in homopolymerization, the copolymerization yields products practically free from unreacted lactide, containing chains composed mainly from poly[(S,S)-lactide] with short end-segments rich in poly(ε-caprolactone) [85].

Polylactides belong to polyesters, which were synthesized in the broadest range of molar masses. Almost all the above-mentioned metal alkoxide-based and metal octoate-based initiators were suitable for the synthesis of polymers with *M*_n_ up to 40,000. There are many reports on polylactides with *M*_n_ exceeding 100,000 that were synthesized using the Sn(Oct)_2_-based initiators. Polylactides with *M*_n_ up to 100,000 were obtained using Al(O^i^Pr)_3_ [86]. Polymer with the highest *M*_n_ approaching 10^6^ was obtained in the polymerization initiated with Sn(OBu)_2_ [87].

Many researchers were concerned about the possibility of the harmful effects of organometallic compounds on the human body. Therefore, there was concern about the use of polymers obtained with initiators containing metals, especially heavy metal derivatives, in medical applications. In general, this topic has not been well studied, except for tin. After extensive research, the US Food and Drug Administration (FDA) approved using for medical purposes polylactides containing up to 20 ppm of tin [60,80].

Concern over the use of polylactides containing heavy metal compounds has led to an interest in the polymerization of lactides with metal-free initiators. One type of these initiators are compounds formed in situ in reactions of alcohols with bases. The term superbase has been adopted for some very strong bases used for this purpose. The first review papers on the metal-free polymerization systems were published about two decades ago [88,89]. Such compounds were used as strong bases as: 4-pyrrolidinopyridine (PPY) [88,89,90], 4-dimethylaminopyridine (DMAP) [88,89,90], 1,8-diazabicycloundec-7-ene (DBU) [91,92,93], 1,5,7-triazabicyclo [4.4.0]dec-5-ene (TBD) [90], N-methyl-1,5,7-triazabicyclo [4.4.0]dec-5-ene (MTBD) [94], phosphines (PBu_3_, PPhMe_2_, PPh2Me, PPh_3_, and chiral phosphines: 1,1′-bis(di-i-propylphosphino) ferrocene, ^®^-[(*S*)-2-(dicyclohexylphosphino)-ferrocynyl] ethyldicyclohexylphosphine, (–)-1,1′-bis(2*S*,4*S*)-2,4-(diethylphospholano) ferrocene, and (–)-1,2-bis(2*R*,5*R*)-2,5-(dimethylphospholano)-benzene) [95], carbenes [96,97], and thiourea-amine [98].

**Figure 6:**
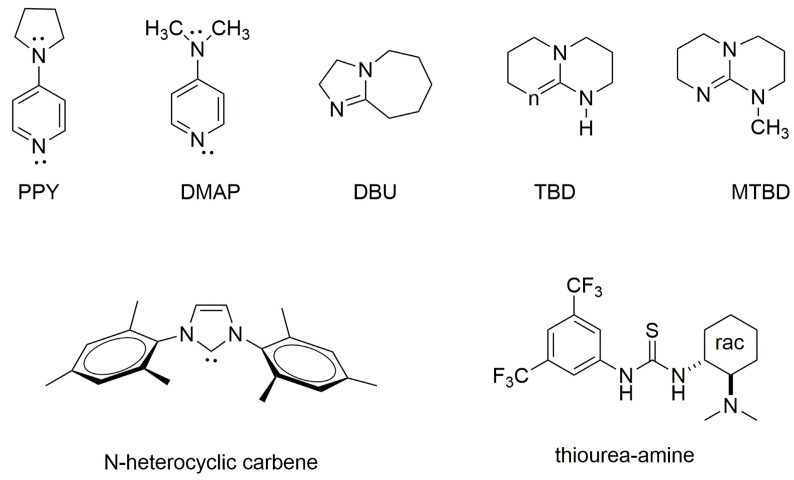
Base components of metal-free initiating systems of the polymerization of lactides: PPY (4-pyrrolidinopyridine), DMAP (4-dimethylaminopyridine), DBU (1,8-diazabicycloundec-7-ene), TBD (1,5,7-triazabicyclo[4.4.0]dec-5-ene), MTBD (N-methyl-1,5,7-triazabicyclo[4.4.0]dec-5-ene), *N*-heterocyclic carbene, thiourea-amine.

**Figure 7:**
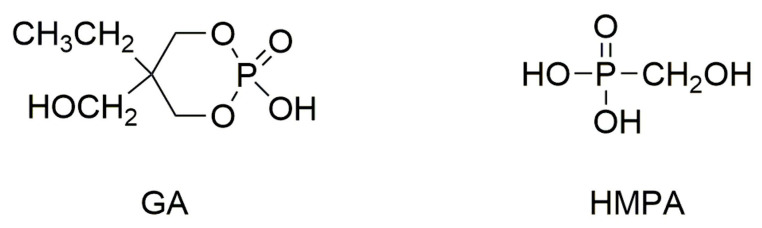
Basic components of the metal-free initiating systems of the polymerization of lactides (GA) and ε-caprolactone HMPA.

Another group of initiating systems consists of a combination of alcohols, which act as initiators, and acids, which function as catalysts leading to polymerization according to the activated monomer mechanism [99,100,101,102]. In polymerization by activated monomer mechanism, the active centers are usually protonated monomer molecules, and propagation involves their addition to the hydroxyl end-groups of the chains. Therefore, in the metal-free propagating systems, neither basic nor acidic catalysts are covalently bonded to macromolecules and therefore can diffuse out from the matrices of polylactide drug carriers and interact in a harmful way with patients’ organisms. Recently, Penczek showed that this problem can be solved by using the initiating systems containing both initiating and catalytic fragments in one molecule [103,104]. The authors use the acronym CINICAT for this class of initiators. Polymerization was initiated using 5-ethyl-2-hydroxy-5-hydroxymethyl-1,3,2-dioxaphosphorinane-2-oxide (GM) and hydroxymethyl phosphonic acid (HMPA), in the case of *S*,*S*-lactide and ε-caprolactone, respectively. The CINICAT moieties were shown to be covalently bound to the synthesized polyester chains as their end groups and did not diffuse out of the polymers. Examples of the structures of base components of the metal-free initiating systems and of CINICAT compounds are shown in Figure 6 and Figure 7, respectively.

**Scheme 6:**
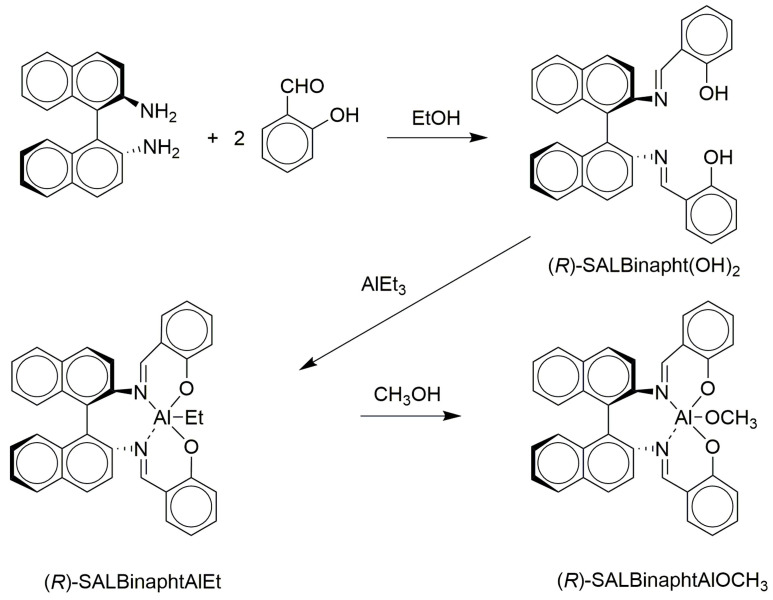
A pathway for the synthesis of ^®^-SALBinaphtAlOCH_3_, an initiator for the stereoelective polymerization of racemic lactide.

Since lactides contain chiral centers in their molecules, some researchers investigated their stereospecific polymerization. While the conversion of pure lactide enantiomers into stereoregular polymers is rather trivial, the attention was mainly concentrated on the polymerization of the racemic mixtures of the (R,R)- and (S,S)-lactides yielding racemic mixtures of the enantiomerically pure homopolymers and/or multiblock copolymers with long stereo sequences. There were also investigated stereospecific polymerization processes, which preferentially yielded polymers with a particular enantiomer chosen from the initially racemic monomer mixture. The first report on the aforementioned stereoelective polymerization of lactide has been published by N. Spassky et al. [105]. The authors obtained a chiral initiator by a three-step process. First synthesized was a chiral Shiff base ligand, ^®^-2,2′-[1,l′-binaphthyl-2,2′-diylbis(nitrilomethylidyne)]diphenol, denoted as ^®^-SALBinapht, in the reaction of ^®^-1,l′-binaphthyl-2,2′-diamine with salicylaldehyde. Then, in reaction with AlEt_3_, the synthesized ^®^-SALBinapht base was converted into ^®^-SALBinaphtAlEt. The last step consisted of a reaction of ^®^-SALBinaphtAlEt with CH_3_OH, producing the ^®^-SALBinaphtAlOCH_3_ initiator. The described synthetic process is illustrated in Scheme 6.

The stereoselectivity of the polymerization of a racemic mixture of (*R*,*R*)-lactide and (*S*,*S*)-lactide initiated with ^®^-SALBinaphtAlOCH_3_ was high, with up to 88% enrichment in *R* constitutional units at 19% of monomer conversion.

**Figure 8:**
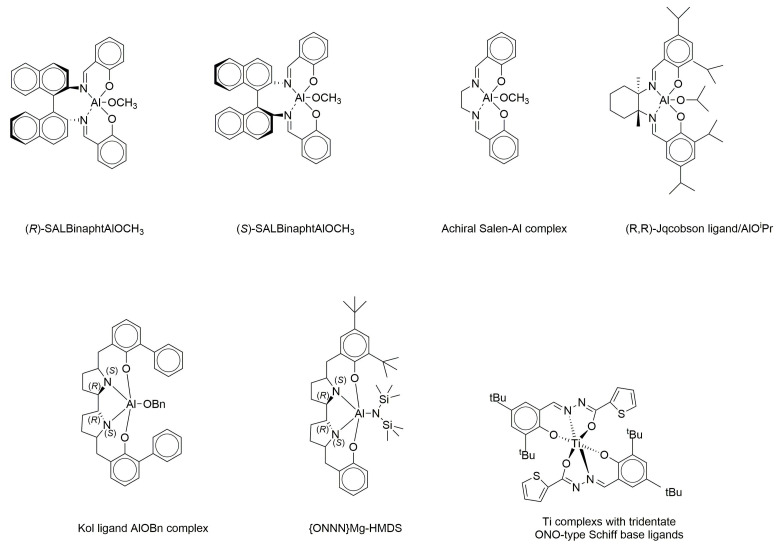
Initiators used for stereospecific polymerization of lactides.

In the following years, ways were developed to synthesize many other initiators containing Schiff base-type moieties and aluminum, zinc, magnesium, titanium, or zirconium alkoxides, useful for inducing stereospecific (stereoselective and stereoelective) polymerization of racemic lactide [106,107,108,109,110,111,112,113,114,115,116,117]. These polymerizations made it possible to obtain polymers with controlled microstructure and thus with controlled degradability required for drug carriers. Examples of these initiators are shown in Figure 8.

**Scheme 7:**
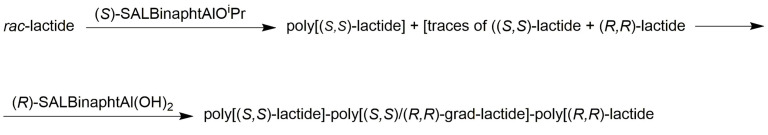
One-pot synthesis of the the poly[(*S*,*S*)-lactide]-poly[(*S*,*S*)/(*R*,*R*)-grad-lactide]-poly[(*R*,*R*)-lactide] copolymer.

Systems that allow ligand exchange during polymerization provide interesting opportunities for the synthesis of enantio-different di-block copolymers from the *rac*-lactide by the one-pot consecutive stereoelective polymerization. An interesting example was presented by A. Duda et al. [118]. In this process, the polymerization of *rac*-lactide was initiated with (*S*)-SALBinaphtAlO^i^Pr (produced in situ in the reaction of (*S*)-SALBinapht(OH)_2_ with Al(O^i^Pr)_3_ trimer), and propagation was carried on until the monomer conversion approached 50%. At this moment, when almost the whole amount of (*S*,*S*)-lactide has been polymerized, an equimolar amount of ^®^-SALBinapht(OH)_2_ was added to the mixture. It is worth noting that the exchange does not have to be quantitative, but because it is reversible, all chains have a chance to be capped with the ^®^-SALBinapht ligand for some time and thus enable the addition of the remaining (*R*,*R*)-lactide. As a result, the poly[(*S*,*S*)-lactide]-poly[(*S*,*S*)/(*R*,*R*)-grad-lactide]-poly[(*R*,*R*)-lactide] copolymer has been produced. The reaction path is shown in Scheme 7. It should be mentioned that despite low molar masses (a few thousand), the melting temperature was very high (210 °C) due to the formation of stereocomplexes between the poly[(*R*,*R*)-lactide] and poly[(*S*,*S*)-lactide] chains.

### 2.3. Polyglycolic Acid (Other Name: Polyglycolide)

Polyglycolic acid (PGA), also called polyglycolide (PGL), is an important polyester whose chains differ from those of polylactide only by the absence of methyl groups. It can be obtained like polylactide by polycondensation of molten glycolic acid or by ring-opening polymerization of glycolide (the glycolic acid cyclic dimer) [119]. The polymer was commercialized in 1970 by DuPont for the production of degradable Dexon^®^ sutures. It is worth mentioning that the USA Drug and Food Administration has approved its use for medical purposes, including drug delivery systems. The glass transition of PGA ranges from 35 to 40 °C, which is close to the temperature of the human body, and melting temperature is in the range 200–225 °C. The PGA degrades relatively quickly. Samples of nearly amorphous PGA (about 5% degree of crystallinity) with 36 mg mass and initial thickness 0.5 mm required about a month for almost complete degradation in phosphate-buffered saline with an initial pH = 7.4 and ionic strength of 0.01 M [120,121]. As degradation progressed, the pH of the liquid phase decreased due to the increased concentration of carboxylic end-groups produced during hydrolysis [120,121].

Except for HFIP (hexafluoroisopropanol), PGA is insoluble in organic solvents. This is a major drawback since all methods used to produce nano- and microcarriers require polymers in solution. This problem has been solved by replacing PGA with glycolide-lactide copolymers (PLGA). There are hundreds of publications on PLGA carriers, mainly on drug-loaded nanofibers [122,123,124]. PLGA has also been used to prepare drug-loaded nanoparticles [125,126,127].

### 2.4. Poly(ε-Caprolactone) and Macrolactones

Poly(ε-caprolactone) (PCL) is a polyester often considered a promising candidate for the preparation of polymeric nano- and microparticle drug delivery systems. Of the polyesters commonly used to make drug carriers, poly(ε-caprolactone) chains contain the longest flexible aliphatic segments separating degradable ester groups. The glass transition temperature (*T*_g_) of PCL reported in the literature ranged from −60 to −40 °C and the melting temperature (*T*_m_) from 58 to 64 °C [128,129,130]. Therefore, particles made of PCL can be manufactured at mild temperatures, eliminating the danger of destroying the encapsulated bioactive compounds. When choosing PCL as a drug carrier, it is important to remember that this polymer degrades slowly and should therefore be used mainly in continuous drug delivery systems. In the human body, PCL is degraded by hydrolysis of the ester groups. The rate of degradation depends not only on the molar mass of PCL but also on the shape and size of the objects being degraded and the local pH. For macroscopic objects, it may need even more than 24 months [131]. However, PCL nanoparticles can degrade faster.

*ε*-Caprolactone was polymerized by cationic, anionic, and coordination processes. The cationic polymerization was carried out according to the activated monomer mechanism [99,132]. In this process, the polymerizing mixture, in addition to the monomer, contains protic acid and growing chains with hydroxyl end groups. At the beginning of the polymerization, the mixture must also contain an alcohol that functions as an initiator. The first step involves protonation of the ester group of the monomer. Then a protonated monomer is added to the initiator (alcohol) or the hydroxyl chain-ends and produces chain-ends with protonated hydroxyls (…-CH_2_OH_2_^+^). Migration of a proton from the charged end-group completes the reaction cycle, extending the polymer chain by one constitutional unit. PCL with *M*_n_ up to 140,000 was obtained by this method [132].

Studies of anionic polymerization initiated by lithium t-butoxide and sodium trimethylsiloxide were carried out in the late 1970s and early 1980s and showed that the process is inevitably accompanied by the formation of cyclic oligomers, whose concentration for polymerization in solution carried out at 0 °C at equilibrium was 0.25 mol/L [133,134,135]. It was assumed that the anionic forms of alkoxides are too reactive and react with ester bonds not only in the monomer but also in the polymer chains (via back-biting). Therefore, less reactive covalent aluminum alkoxides, tin (II) alkoxides, tin(II) bis(2-ethyl hexanoate) (Sn(Oct)_2_) derivatives (Sn(Oct)_2_/ROH and SnOct)_2_/RNH_2_) were used in later studies. First, it was shown that the polymerization of ε-caprolactone initiated with CH_3_OAl(CH_2_CH_3_)_2_ is effectively suppressed [136]. Later, it was observed that the reactivity-selectivity principle is general in the polymerization of ε-caprolactone and that …-OC(O)(CH_2_)_5_OAl(^i^C_4_H_9_)_2_, albeit much less reactive than …-OC(O)(CH_2_)_5_O-Na+ are more reactive in propagation than in back-biting (ratio of propagation to the back-biting rate constant k_p_/k_b_ = 7.7 × 10^4^ L/mol), practically eliminating cyclic oligomers from the synthesized PCL [137]. The polymerization of ε-caprolactone initiated with ^i^PrOAl(OCH_3_)_2_ yielded polymer with *M*_n_, 430,000 [138].

**Figure 9:**
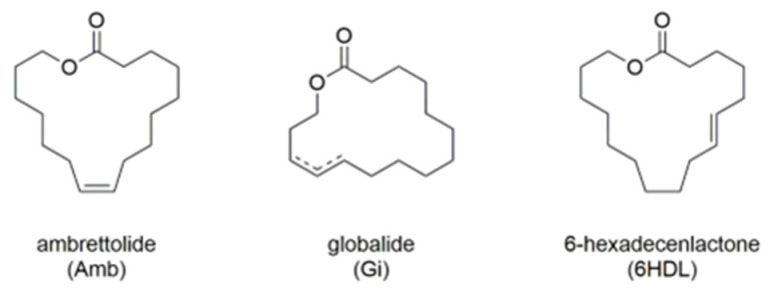
Macrolaactones containing double bonds in the ring.

Polyesters commonly used to make drug carriers typically lack groups that would allow them to be functionalized on demand and in a controlled manner. Therefore, recently macrolactones containing double bonds have attracted the attention of researchers who have used them as comonomers in the copolymerization of ε-caprolactone [139,140,141]. Examples of these macrolactones are shown in Figure 9.

It should be noted that macrolactones containing a double bond have been effectively incorporated into PCL chains. The copolymers can be used as an additive that, when dissolved together with standard PCL, can be used to prepare carriers equipped with double bonds suitable for functionalizing them in the thiol-ene reaction [139,140].

## 3. Preparation of Polyester Drug Carriers

**Figure 10:**
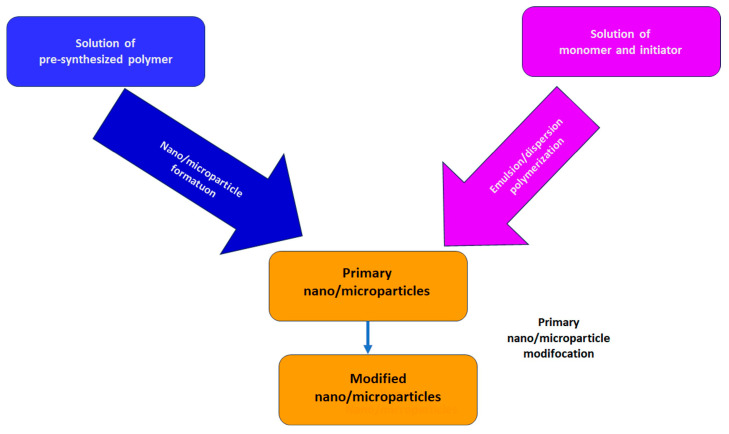
Main strategies for obtaining polyester drug carriers: from previously synthesized polymers and directly from monomers by polymerization.

There are two major strategies and several methods for the preparation of polyester carriers of drugs and other therapeutic compounds (for example, compounds used for diagnostic purposes). These strategies are schematically presented in Figure 10.

Some strategies and methods are general and have been used to prepare nano- and microcarriers from many types of polymers. However, some are specific to polyester carriers. However, they all have one thing in common—they are solution-based processes.

The most common strategy is based on preparing drug carriers from previously synthesized polymers. It involves a series of methods, sometimes quite complex, that ultimately transform polymer pieces into nano- or microparticles of the required size. Since ways to mechanically disintegrate solid polymer into nano- and microparticles of the required and uniform size are unknown, simpler methods involving dispersing a polymer solution in the gas or liquid phase and then solidifying the formed particles have been used. Polymer solutions containing the drug allow the preparation of polymer particles loaded with the drug.

The second strategy involves dispersion or emulsion polymerization of suitable monomers. Very often, the original particles require further modification before they can be used for drug formulation.

Methods of preparing nano- and microcarriers and their advantages and disadvantages are discussed below.

### 3.1. Drug Carriers by Oil-in-Water (O/W) Method

A method called “oil in water” is used to prepare particles containing hydrophobic bioactive compounds. This is a general method that is not specific only to polyester particles but is also suitable for other particles made of hydrophobic polymers. First, the polymer and drug are dissolved in an organic solvent with a low boiling point and miscible with water to a few percent. The polymer and drug solution is gradually added to water, which may contain a surfactant and may contain a hydrophilic polymer (such as polyvinyl alcohol (PVA)). The mixture is effectively homogenized or otherwise blended. Methylene dichloride is often used as an “oil.” Dispersion produces nano- or micro-droplets of “oil” (organic solvent) containing the polymer and drug. Migration of the organic solvent through the water-based solution toward the air and its eventual evaporation transforms the droplets into solid particles suspended in a water-based continuous phase. The crude product is often purified by isolating it several times by centrifugation-resuspension in an aqueous continuous phase. The O/W method has been successfully used to prepare drug-containing particles from polyhydroxybutyrate (PHB), polylactides (PLA), poly(lactide/glycolide) (PLGA), poly(ε-caprolactone) (PCL), and copolymers containing blocks of the above-mentioned polyesters. The O/W method is probably the oldest used to obtain polymeric drug carriers. Therefore, it may be surprising how many interesting and important improvements have been made over the past fifteen years. As a result, the O/W method has been adapted to produce polyester particles with average diameters ranging from 60 nm to ≈50 μm [142,143,144,145,146,147,148,149,150]. It is worth mentioning that particle diameters are of great importance for drug carriers, as they affect their distribution in the body and uptake by cells [126,151]. Some studies have yielded unexpected results. For example, it was shown that appropriate adjustment of the viscosity of the “oil” and “water” (containing polyvinyl alcohol) phases, the mixing rate, the molar mass, and the structure of the end groups of the copolymer constituting the drug carrier (PLGA) made it possible to obtain drug-free and drug-loaded (paclitaxel) spheroidal PLGA particles [152]. The results of studies on obtaining PLGA particles using vitamin E as an emulsifier also have great potential [147]. The advantage of vitamin E is that it is a natural substance (containing tocopherols and tocotrienols) that can replace synthetic emulsifiers. In the European Union, vitamin E is approved as a common food additive. Its use has made it possible to obtain PLGA nanoparticles up to about 60 nm in diameter.

Recently, a simple O/W method was developed to prepare indented PLGA microparticles containing encapsulated budesonide, a drug used in the treatment of chronic asthma and other obstructive pulmonary diseases [150]. The final product was obtained by lyophilizing a suspension of the frozen particles and applying it using a standard powder inhaler. The advantage of the method mentioned above is the simplicity of the required particle preparation equipment and better aerodynamic performance than with smooth surface carriers.

### 3.2. Drug Carriers by Water-in-Oil-in-Water (W_1_/O/W_2_) Method

The water-in-oil-in-water (W_1_/O/W_2_) method, which is an extension of the O/W procedure, is designed to prepare water-soluble carriers for bioactive compounds, particularly nucleic acids, oligonucleotides, proteins, and oligopeptides. “Water” refers to a water-based ingredient (often a buffer) that is a solvent for the bioactive compound but is not a solvent for the polymer that makes up the carrier body. The subscripts “1” and “2” indicate that the water-based ingredients can be different. “Oil” refers to an easily evaporated organic liquid that mixes slightly with water and is a good solvent for the polymer but not for the drug. The two phases may contain various surfactants, and the “water” may contain a small amount of hydrophilic polymer (such as PVA). The entire process begins with dissolving the drug in the “water” and dispersing this solution in the “oil” (e.g., in dichloromethane). The resulting W/O mixture consists of a continuous “oil” phase containing the dissolved polymer (e.g., PLGA) and very small droplets of “water” containing the drug. In the next step, the mixture is dispersed in “water”. The product is a mixture of an aqueous continuous phase containing microdroplets of “oil” with nanodroplets of an aqueous phase containing the dissolved drug. The low boiling “oil” component diffuses through the aqueous continuous phase and evaporates. Its elimination transforms microdroplets into drug-containing microparticles. The W/O/W method has been used to obtain nano- and/or microparticles from PLGA [153,154] and PCL [155,156] polymers. Some interesting examples are presented below.

G. Golomb et al. described the preparation of PLGA particles containing an encapsulated human placental alkaline phosphatase gene cloned in the pcDNA3 vector [153]. The particles were produced by a standard W/O/W procedure, using buffers as the “water” phase and chloroform as the “oil.” This work is worth mentioning because it represents the first case of sustained gene delivery allowing pDNA release for about a month.

Antigens used in vaccine production are often water/buffer-soluble proteins or oligopeptides that can be conveniently encapsulated by the W_1_/O/W_2_ method. Therefore, the development of a process suitable for modifying the aforementioned molecules in a way that allows their uptake by dendritic cells and macrophages has been much appreciated. Such a method recently developed for PLGA particles involved conjugation of PLGA particles coated with polydopamine to exosomes and ovalbumin [129]. It is worth mentioning that PLGA nanoparticles conjugated with exosomes were more efficiently taken up by antigen-presenting cells.

Most of the polyester nano- and microparticles produced by the W_1_/O/W_2_ double emulsion method were obtained from linear homo- and copolymers, and the interest in the influence of the copolymer architecture on their assembly and drug loading is understandable. Therefore, it is worth mentioning the study of particles obtained from six-arm copolymers with poly(ε-caprolactone)-poly(ethylene oxide) arm structure (6S-PCL-PEO) [155]. This paper presents the preparation and characterization of model particles loaded with ovalbumin (OVA). The average diameter of the obtained unloaded nanoparticles was about 191 nm. The diameter of nanoparticles with encapsulated OVA was slightly larger (233 nm). Nanoparticles with such small diameters are well “visible” to macrophages. The authors of the above study have provided a model system that, when compared with nanoparticles from linear copolymers with similar molar mass ratios of PCL and PEO blocks, could reveal the influence of the polymer architecture on the nanoparticle properties. Recently, R.D. Gökberk et al. developed a pulmonary drug delivery method based on PCL particles produced by a W_1_/O/W_2_ double emulsion process [156]. The preparation of PCL particles containing lysozyme, an antimicrobial peptide, was optimized to provide high encapsulation efficiency (EE 65.15%). Their median aerodynamic parameter was 5.44 ± 0.19 μm, and the fine particle delivery fraction (fraction of particles with less than 5 μm in diameter) was 51.0 ± 2.9%. The long-term release of lysozyme from particles lasting up to 35 days is very important. The above formulation can be considered as an alternative to antibiotic-based systems.

A disadvantage of hydrophilic drug particles prepared by the W_1_/O/W_2_ method is the often-observed initial rapid release of the drug. Recently, this disadvantage has been alleviated by converting a hydrophilic drug (e.g., bovine serum albumin—BSA—used as a hydrophilic drug model) into a hydrophobic drug [157]. The “hydrophilic-hydrophobic” conversion was carried out by mixing an aqueous solution of BSA with a tert-butanol solution of lectin; the mixture was frozen and lyophilized. The resulting BSA-lectin (S) nanoparticles were easily dispersed in dichloromethane “oil” containing dissolved PLGA. This dispersion was transformed into BSA-loaded PLGA particles, similar to the second O/W_2_ step of a typical W_1_/O/W_2_ process. The entire modified process, which can be referred to as W_1_/S/O/W_2_, yielded nanoparticles with a significantly reduced initial burst.

### 3.3. Drug Carriers by Nanoprecipitation

Nanoprecipitation is a process involving two mutually miscible liquids (at least in certain proportions). The first liquid should be a solvent for the polymer and the drug (e.g., acetone). The second liquid (e.g., water) should be a non-solvent for the polymer and preferably also a non-solvent for the drug, but the latter requirement is not crucial. Both liquids can also contain surfactants. Mixing these liquids results in the formation of nanodroplets of the first liquid containing the polymer and the drug, with accompanying deposition of the polymer at the interface. The solvent rapidly diffuses into the non-solvent phase, and this displacement results in the almost immediate formation of drug-loaded polymer nanoparticles. This method was developed by Fessi et al. and used to prepare indomethacin-loaded poly(*R*,*S*-lactide) nanoparticles [158]. It should be noted that there are not only reports describing cases in which the polymer and drug solution were added to the insolvent but also many cases in which the non-solvent was added to the polymer and drug solution [158,159]. It is noteworthy that the first system (acetone/water) mentioned earlier has almost become the standard and is still widely used after many years. In the review, however, we draw readers’ attention to its recent modifications.

For the preparation of nanoparticle drug carriers by nanoprecipitation, all kinds of typical aliphatic polyesters were used, including poly(*R*,*S*)-lactide-*co*-glycolide, poly*(S*,*S*)-lactide-*co*-glycolide, poly(S,S-lactide), poly(*ε*-caprolactone), poly(3-hydroxybutyrate), and poly(3-hydroxybutyrate-*co*-3-hydroxyvalerate) (PHBV). However, while the most comprehensive studies have been conducted for polylactides and related polymers, the least studied (only one paper [160]) have been nanoparticles containing butyric acid ester segments. Here are some examples indicating progress in the controlled preparation of nanoparticles by nanoprecipitation.

In some cases, combination treatment is required with two active compounds delivered in the required proportion to the tissue in question. Such treatment is often desirable for patients suffering from certain cancers. The solution is to prepare double-loaded nanoparticles. Bandyopadhyaya et al. developed the preparation of PLGA nanoparticles with encapsulated curcumin (CUR) and niclosamide (NIC) and compared their properties with those of similar but single-drug-loaded nanoparticles. The nanoparticles were produced by the classical nanoprecipitation method, using acetone as the organic solvent for PLGA, CUR, and NIC and PVA-containing water as the nonsolvent [160]. The average diameters of the doubly charged nanoparticles were 257 nm. The diameters of not loaded nanoparticles only-CUR and only-NIC loaded were slightly smaller (207, 214, 216, respectively). The loading efficiency for doubly drug-loaded nanoparticles was higher than for single drug loading. The authors noted that the advantage of the doubly drug-loaded PLGA-CUR-NIC nanoparticles was their more efficient uptake by cancer cells.

Dual loading is also beneficial for the oral use of drugs sensitive to low pH. Loading the nanoparticles with the drug and a pH stabilizer (such as magnesium oxide used as an alkalizer) can protect the drug from degradation in the stomach. In this way, PLGA nanoparticles loaded with lansoprazole were prepared for prolonged release after oral administration [161].

Classical nanoprecipitation was developed and adapted for the encapsulation of hydrophobic drugs, which for many years was an obvious limitation of this method. Recently, a simple modification has been introduced to enable the use of nanoprecipitation of hydrophilic bioactive compounds. The modified nanoprecipitation has been verified for the encapsulation of water-soluble ciprofloxacin (a drug used in ophthalmic therapy) in poly[(*R,S*)-lactide]-dextran (PLA-DEX) or poly(lactide-*co*-glycolide)-polyethylene glycol (PLGA-PEG) [162]. DMSO was used as a solvent for the aforementioned hydrophilic polymers, and HCl/water was used as a solvent for ciprofloxacin and a non-solvent for PLA-DEX and PLGA-PEG. Nanoparticles were obtained by adding a DMSO solution of the polymers to an aqueous HCl solution of ciprofloxacin at a controlled rate. The diameters of the particles obtained ranged from 82 to 205 nm, depending on the polymer used and the loading of the nanoparticles with the drug. The maximum mass loading of the drug reached 27.24%.

### 3.4. Preparation of Drug Carriers Using Spray-Drying Equipment and Chips Used in Microfluidic Techniques

The above subsections describe the physicochemical processes used to produce polymeric drug nanocarriers. This one describes the use of commercially available equipment for this purpose.

Some diseases require direct treatment of the lung tissue. In such cases, the method of choice is treatment with dry powder preparations administered by inhalation. Drug preparations in dry powder form are usually prepared using spray dryers of a design similar to equipment commonly used in the food industry to produce powdered milk or instant coffee. For laboratory purposes, equipment is sold that allows the preparation of inhalation formulations on a scale of a few grams. Each spray dryer consists of the following parts: an atomizer powered by a polymer and drug dissolved preferably in a volatile solvent; a drying chamber powered by the fine droplets produced by the atomizer and connected to a source of hot air or nitrogen that transforms the droplets into particles and transports them to a cyclone where a vortex of gas directs them to a collector [163]. Traces of the remaining particles suspended in air or nitrogen flow toward the filters and are eventually expelled. The main advantages of spray-drying nanoparticle manufacturing are its versatility, tunability, simplicity, and scalability. Disadvantages include exposure of bioactive components to hot air, the high pumping capacity required, and the large volume of liquid (often organic) to be evaporated and recycled. Moreover, it is worth noting that the diameters of polyester particles produced by spray drying typically exceed the nanometer range. For example, the diameters of PLGA particles containing Artemisone (an antimalarial drug) range from 1 to 1.7 μm [164].

Microfluidic techniques are based on microreactors consisting of various microchannels and reaction chambers of fixed or variable arrangement. They differ from the usual laboratory glassware used to produce nanoparticles (such as beakers, flasks, stirrers, rotary evaporators, etc.) by their working volume, which is much smaller. The parameters of particles produced in microfluidic reactors depend not only on the design of these devices. For example, when particles are formed by injecting a solution of a polymer and any other bioactive component dissolved in an organic solvent (e.g., in dichloromethane) into a containing water microreactor, the fine droplets are formed and solidify into microparticles. The diameters of particles depend on the flow rate of the organic and aqueous phases and the inner diameter of the injection needle. A device with an inner needle diameter of 1.3 mm yielded microparticles with diameters ranging from 130 to 700 μm. Large microparticles were suitable for the embolization of capillary blood vessels in the treatment of early-stage aneurysms [165].

Drug nanocarriers are known to be produced by combining two or even three methods in a single process. The above approach was used to prepare PLGA particles loaded with niclosamide and equipped with a hydrophilic coating of poly(vinyl alcohol) PVA and hyaluronic acid (HA) [166]. Niclosamide is a multifunctional drug used to treat cancer, metabolic disorders, and viral infections. It is insoluble in water and, as a solid, is highly hydrophobic. As such, it must be encapsulated in nanoparticles coated with hydrophilic compounds to be dispersed conveniently. The first step of the process involves dissolving equal amounts of PLGA and niclosamide in an appropriate volume of ethyl acetate (“oil” phase). In parallel, a known volume of aqueous PVA solution (aqueous phase) is prepared. The two phases are mixed until a homogeneous mixture is obtained. Then a certain amount of hyaluronic acid is added to the mixture, which is injected into the microfluidic system in the next step and further mixed slowly. The mixture is then subjected to ultrasound and stirred until the ethyl acetate evaporates, accompanied by particle formation. In summary, the entire process combined the oil-in-water method using classical mixing and stirring in a microfluidic system, with a final treatment involving ultrasound, dialysis, and lyophilization.

Recently, Schneider et al. described an example of multidrug-carrying nanoparticles prepared by elegantly combining nanoprecipitation in a microfluidic microreactor with a spray-drying technique [167]. Primary PLGA nanoparticles containing curcumin were obtained by nanoprecipitation, injecting a solution of the drug and PLGA dissolved in acetonitrile and an aqueous solution of Pluronic F68 into a microfluidic reactor. The optimized process yielded nanoparticles with a diameter of 105 nm. Spray drying of combined PLGA-curcumin nanoparticles and antibiotics (tobramycin, ciprofloxacin, or azithromycin) and N-acetylcysteine (NAC) yielded inhalable formulations with a median mass aerodynamic parameter (MMAD) ranging from 2.16 to 2.63 μm and a fine particle fraction (FPF; fraction of particles with MMAD < 5 μm) ranging from 61.6 to 97.5%.

### 3.5. Direct Synthesis of Polyester Nano- and Microparticles by Ring-Opening Dispersion Polymerization of Lactides and ε-Caprolactone

Direct synthesis of particles has the following advantages: easy scalability, similar to dispersion polymerization of vinyl monomers; easy adjustment of particle diameters and molar masses of polymers to requirements; narrow dispersion of diameters and molar masses; and control of interfacial properties of particles. The first syntheses of nano- and microparticles of polylactide and poly(ε-caprolactone) were carried out in 1994 by Slomkowski et al. [168]. Dispersion polymerization of *ε*-caprolactone was initiated using diethylaluminum ethanolate and carried out in the presence of poly(dodecyl acrylate)-g-poly(ε-caprolactone) surfactant in a mixed 1,4-dioxane-heptane continuous phase (1:8 *v*/*v*). Poly[(*R*,*S*) lactide] microspheres were prepared by a process with tin (II) octanoate used as catalyst/initiator and poly(dodecyl acrylate)-*g*-poly(*ε*-caprolactone) added as surfactant. The continuous phase was a mixture of 1,4-dioxane and heptane in a ratio of 1:4 *v*/*v*. The average diameters (*D*_n_) and diameter dispersities (*Đ*_D_ = *D*_v_/*D*_n_) were 0.628 μm and 1.38, and 1.25 μm and 1.15 for poly(*ε*-caprolactone) and polylactide particles, respectively. Subsequent publications provided comprehensive information on the kinetics and mechanism of dispersion polymerization used to better control particle properties. It was noted that the dispersity of directly synthesized poly[(*S*,*S*)-lactide] microspheres strongly depended on the concentration and composition of the surfactant poly(dodecyl acrylate)-g-poly(*ε*-caprolactone) [169,170]. The best results (the dispersion with the smallest diameter, *χ* = 1/(*Đ*_D_-1) = 33; *Đ*_D_ = 1.03) were obtained for the ratio of *M*_n_ of poly(ε-caprolactone) strains and *M*_n_ of the whole surfactant macromolecule equal to 0.23 [145]. For poly(dodecyl acrylate)-*g*-poly(*ε*-caprolactone) with an average number of strains of 1.3 (*M*_n_ = 4700) *Đ* = 1.08 [169]. A summary of the most important results gives a very simple picture of the dispersive ring-opening polymerization of *ε*-caprolactone and lactides [170,171,172,173]. At the very beginning of polymerization, the system is homogeneous and contains 1,4-dioxane and heptane in the appropriate proportion, a monomer (*ε-*caprolactone or lactide), a surfactant [poly(dodecyl acrylate)-*g*-poly(*ε*-caprolactone)], and an initiator (diethylaluminum ethanolate or tin (II) octanoate). Initiation takes place in solution, and the chains begin to grow in solution as well. When the growing chains reach a certain critical length, they undergo a chain-globule transition and aggregate into particle nuclei. In *ε*-caprolactone polymerization, this process takes about 100 s. After this initial period, all active centers participate in propagation inside the particles, which are supplied with monomers from the continuous phase. It has also been noted that in the polymerization of ε-caprolactone and any of the lactides, the number of nanoparticles initially formed during nucleation remains constant at later stages, i.e., neither new particles are formed nor do they aggregate [170,171,172]. As a result, in the ring-opening dispersion, polymerization particles should be treated as nano or microreactors containing all active propagating species and a significant part of the ε-caprolactone or lactide monomers. Because the volume of particles is much smaller (in the majority of experiments, ca. eight times) than the whole volume of the polymerizing mixture (the volume of particles + the volume of continuous phase), the local concentration of monomers and propagating species is higher, and the rate of polymerization should be higher than the otherwise analogous polymerization in solution. Indeed, it was noticed that rates of polymerization in the dispersed systems (i.e., in 1,4-doxane/heptane mixtures) were about ten times higher than rates of polymerization in solution (in THF) [170,172]. At the end of polymerization, the propagating centers were deactivated by the addition of a small amount of acetic acid. The particles were purified by the several times repeated cycle of sedimentation-isolation-resuspension in fresh portions of heptane. Heptane was also a medium for their storage.

In later years, several new surfactants were synthesized: poly(*ε*-caprolactone)-*b*-poly(dodecyl acrylate), poly(*ε*-caprolactone)-*b*-poly(octadecyl methacrylate)-*b*-poly(dimethylaminoethyl methacrylate), poly{(dodecyl methacrylate)-*co*-poly{α-methacryloxyethoxy-poly{(*S*,*S*)-lactide}}}, poly{(dodecyl methacrylate)-*co*-poly{α-methacryloxyethoxy-poly{(*R,S*)-lactide}}}, poly[(dodecyl methacrylate)-*co*-(hydroxyethylmethacrylate)], poly[(dodecyl methacrylate)-*g*-poly[(*R*,*S*)-lactide] [126,174,175,176]. Their use has not led to the development of a fundamentally new mechanism for the dispersion polymerization of cyclic esters. However, it is worth noting that the use of poly[(dodecyl methacrylate)-*co*-(hydroxyethyl methacrylate)], which undergoes in situ transformation into poly[(dodecyl methacrylate)-*co*-(hydroxyethyl methacrylate)-*g*-poly[(*R*,*S*)-lactide], simplified the synthesis [175]. Poly(*ε*-caprolactone) and polylactide are synthesized by dispersion polymerization and form stable suspensions in organic media. However, for use as drug carriers, they should form stable suspensions in water, buffers, and other water-based media. The method developed consisted of replacing the suspending medium (heptane) with an ethanol-KOH solution containing surfactants. Controlled hydrolysis time allowed to obtain particles with hydroxyl and carboxyl groups in the interfacial layer, providing additional electrostatic stabilization for stabilization by surfactants. The range of pH and ionic strength at which suspensions of poly(ε-caprolactone) and poly[(S,S)-lactide] are colloidally stable has been determined [177].

### 3.6. Preparation of the Directly Synthesized Polyester Nano- and Microparticles Loaded with Drugs or Drug Models

The use of the strategies listed below to prepare directly synthesized drug-loaded particles has been verified for the following methods:

Adsorption or covalent immobilization of selected compounds on the particle surface. Examples include the adsorption of human serum albumin (HSA) and *γ* globulin (γG) on poly(*ε*-caprolactone) and poly[(*R*,*S*) lactide] particles [168]. In the case of poly(*ε*-caprolactone), the maximum surface concentration of adsorbed protein (at saturation) was 1.0 mg/m^2^ and 2.6 mg/m^2^ for HSA and *γ*G, respectively. For adsorption on poly[(*R*,*S*) lactide] molecules, the maximum surface concentration was 0.9 mg/m^2^ and 1.7 mg/m^2^ for HSA and γG, respectively. Another example is the covalent immobilization of 6 aminoquinoline (6AQ) on the surface of poly[(*S*,*S*) lactide] microspheres. The maximum surface area of covalently immobilized 6AQ was 0.27 mg/m^2^ [177].

Very simple is loading polyester particles with a hydrophobic, liquid drug able to swell the carriers. For example, the poly(*ε*-caprolactone) particles with diameters *D*_n_ = 620 nm were suspended in a 70%/30% *v*/*v* ethanol/water mixture. To portions of this suspension, a varied amount of ethyl salicylate was added. The concentration of nanoparticles was 5.4 mg/mL in all samples. The concentration of ethyl salicylate ranged from 1.7 mg/mL to 56.6 mg/mL. The samples were shaken at room temperature for 48 h. The particles were separated by centrifugation, and the content of the encapsulated drug was calculated as the difference between its concentration in the continuous before and after incubation. The particles with a degree of loading in the range from 9.7 to 37.0 wt% were obtained [177]. Higher loading was also possible, but such particles were converted to droplets of viscous liquid.

The use of bioactive compounds containing hydroxyl groups as transfer agents in the dispersion polymerization of cyclic esters has proven suitable for the direct synthesis of drug-loaded nanoparticles. For example, the polymerization of ε-caprolactone ([ε-caprolactone]_0_ = 3.8 × 10^−1^ mol/L) initiated by (CH_3_)_3_SiONa ([(CH_3_)_3_SiONa]_0_ = 1.76 × 10^−3^ mol/L) and carried out in a 1,4-dioxane/heptane 1:9 *v/v* mixture in the presence of *N*,*N*-bis(2-hydroxyethyl)isonicotinamide (transfer agent; [*N*,*N*-bis(2-hydroxyethyl)isonicotinamide]_0_ = 2.2 × 10^−2^ mol/L) and poly(dodecyl acrylate)-*g*-poly(*ε*-caprolactone) (surfactant; 1.6 g/L) yielded poly(*ε*-caprolactone)/*N*,*N*-bis[poly(*ε*-caprolactone)]isonicotinamide nanoparticles with a drug content of 6.4 wt%.

Direct entrainment of 5-methoxy-2-{[(4-methoxy-3,5-dimethyl-2-pyridyl)-methyl 1]-sulfinyl}-1H-benzimidazole (omeprazole) during the synthesis of poly[(*S*,*S*)-lactide] nanoparticles by ring-opening dispersion polymerization, it was used as a simple method to obtain polyester drug carriers [178]. The polymerization was carried out as described in Section 3.5 with one small modification. Since the nucleation of the particles was completed, omeprazole (an inhibitor of (H*-K^+^)ATPase, the “pH pump” responsible for the secretion of HCl in the stomach) was gradually added. The following initial reagent concentrations were used in the synthesis: [(*S*,*S*)-lactide]_0_ = 2.5 × 10^−1^ mol/L, [tin (II) 2-ethylhexanoate]_0_ = 4.6 × 10^−3^ mol/L, [poly(dodecyl acrylate)-*g*-poly(*ε*-caprolactone)]_0_ = 1.6 × 10^−1^ g/L. After adding the appropriate amount of omeprazole, the total concentration of the drug in the polymerization mixture was 5 × 10^−3^ mol/L. The percentage of omeprazole in the produced particles was 11% [179].

## 4. Targeted Drug Delivery to the Brain Based on Polyester Nanoparticles

There is a long list of active substances that, as drugs, can be administered orally in tablets or loaded into macroscopic capsules, for which it is only required to pass from the gastrointestinal tract into adjacent blood vessels and be distributed throughout the body with the blood. Typical examples include soya lipids to support liver function, progesterone (lutein) often used by patients with vision problems, and even such simple compounds as KCl prescribed to many cardiac patients. However, for many drugs, simple systemic administration of untargeted drugs is undesirable. This is the case, for example, when systemic administration causes undesirable side effects by placing the drug primarily in an organ other than where it should act or does not allow the drug to reach its chosen site of action. This section presents the main types of polyester carriers for targeted drug delivery to selected parts of the brain or to particular types of brain cells.

The brain is a very delicate organ that requires effective mechanical protection. Moreover, it can function properly only in a suitable, chemically stable environment and should not be directly exposed to uncontrolled electromagnetic stimuli. The skull usually provides sufficient mechanical protection. Since bones are not permeable by water-based liquids, the skull also protects the brain from uncontrolled interactions with body fluids. However, the brain must maintain some contact with the environment, from which nutrients are supplied and into which unwanted metabolites are removed. The brain must maintain the information exchange channels provided by nerves. The nutrients are transported in the blood vessels crossing the scull. However, the endothelium of these vessels is so tight that it creates the blood-brain barrier, which only some compounds can overcome [7,8]. The nerves used for an exchange of information transport electric signals and as a route for the transportation of medically active compounds can be used only in very special instances such as nasal drug administration. This is because the nasal cavity contains the only location (the so-called olfactory region), where the nerves of the central neural system present in the mucosa are exposed to external contacts. Unfortunately, the surface of the olfactory structures in humans is quite small and approaches 0.3 m^2^ (taking into account microvilli) [180], which limits the nasal transport potential. However, the research on using the aforementioned methods continues.

There are some life-threatening illnesses, which should be considered manifestations of structural and functional disorders of parts or of the whole brain. The hopes of their cure or at least alleviation of symptoms depend on finding the appropriate drug and its targeting carrier to the needed site.

As the first example, there are presented carriers for treatment of the brain-degenerating Parkinson’s disease. Parkinson’s disease is manifested by such symptoms as tremors, rigidity, bradykinesia, and postural instability. All of them are caused by low dopamine levels in the striatum that control movement and muscle function [181,182]. Dopamine is a product of dopaminergic neurons in the part of the brain called the substantia nigra pars compacta, and regardless of the reason for their lesion, the production of dopamine is decreased. It should be noted that the metabolism of dopamine also includes a tract involving its oxidation by monoamine oxidase type B, known for producing hydroxyl radicals harmful to dopaminergic neurons. Therefore, for the Parkinson’s disease-ill patients, it was desirable to block this tract. A few years ago, N. Ahmad investigated the possibility of using rasagiline, a compound known for the deactivation of monoamine oxidase type B [182]. The rasagiline containing PLGA nanoparticles was designed for nasal delivery. They were prepared by the double emulsification-solvent evaporation method. The particles were coated with chitosan to make them mucosa adhesive. The bare PLGA-rasagiline and chitosan-coated (chitosan-PLGA-rasagiline) nanoparticles were comprehensively characterized by providing their diameters and diameter distributions, zeta potential, drug loading, and in vitro drug release. Ex vivo nasal mucosa permeation and brain pharmacokinetics studies for the rat model revealed that the chitosan-PLGA-rasagiline nanoparticles can be effectively used for rasagiline delivery to the brain using the olfactory pathway. Recently, S. Lee et al. reported on using the resveratrol-loaded PLGA nanoparticles for the protection of the dopaminergic neurons of the substantia nigra pars compacta [183]. The lactoferrin-conjugated PLGA-resveratrol nanoparticles were used for this purpose since lactoferrin conjugation enhances the internalization of nanoparticles into the brain microvascular endothelial cells and makes the crossing of the blood-brain barrier more effective. Indeed, the bioluminescent imaging analyses revealed the accumulation of the lactoferrin-PLGA-resveratrol nanoparticles in the brain higher than the accumulation of the bare PLGA-resveratrol nanoparticles.

There are two potentially complementary approaches for the treatment of patients suffering from Parkinson’s disease. The one described above focused on the delivery of drugs protecting the dopaminergic neurons from degradation, and the second one was based on the delivery of the dopamine to the brain, aiming at alleviating the symptoms related to its insufficient level. Nanoparticles used to deliver dopamine to the brain were similar to nanoparticles used to deliver compounds that protect dopaminergic neurons. However, it is worth mentioning the research of R. Pahuja et al., who developed PLGA-dopamine nanoparticles that, despite not containing any targeting molecules, were able to deliver dopamine effectively to the brain [184]. The nanoparticles were prepared by the double emulsification solvent evaporation method (W/O/W). First, dopamine in water was emulsified in dichloromethane. Then this emulsion was emulsified in water containing poly(vinyl alcohol) (PVA), which was used as a stabilizer of nanoparticles’ suspension. Interactions of the dopamine-loaded nanoparticles with the model SH-SY5Y cells (in vitro studies) and with brain cells (in vivo studies using the Parkinsonian rat model). The studies revealed that the dopamine-loader nanoparticles administered intravenously are effectively delivered to the rat’s brain and that this treatment reversed neurochemical and neurobehavioral symptoms in the Parkinsonian rats. Positive results were obtained also using the dopamine-loaded PLGA nanoparticles bearing the lactoferrin and borneol functions [185] and a much more complex albumin/PLGA-dopamine nanosystem [186].

Another illness related to the degeneration of the brain is Alzheimer’s disease. Its early symptoms showing some problems with memory are usually not alarming. Rare difficulties with recalling names of people or places occur almost to everybody. However, in the case of Alzheimer‘s disease, the difficulties increase with time, and suffering people do not recognize their friends and family or are disoriented about time and places. Eventually the patients have problems with speech, changing positions, eating, and swallowing. The structure of their brains is very different from that of healthy people. They contain amyloid beta (Aβ) aggregates, which are toxic to neurons. Alzheimer’s disease is incurable, and its etiology is not known. The only available treatment is based on protection of neurons against their degradation. The only drug approved for this purpose, both in the US and Europe, is memantine. However, the effectiveness of its direct use has been quite low. Several years ago, E. Sánchez-López et al. developed PLGA-based nanocarriers with the hope of improving the transport of memantine across the blood-brain barrier [187]. The memantine-loaded PLGA-PEO nanoparticles were prepared by the double emulsification W/O/W method. During the first step, the memantine was dissolved in deionized water. Then, this solution was emulsified in ethyl acetate containing dissolved PLGA-PEO copolymer. Eventually, the primary emulsion was emulsified in water containing the poly(vinyl alcohol) stabilizer. Evaporation of the liquid organic phase (ethyl acetate) produced a stable suspension of the memantine-loaded nanoparticles dispersed in the water continuous phase. The control memantine-free nanoparticles were produced similarly but without memantine addition. The fluorescent nanoparticles containing rhodamine were also prepared for the biodistribution studies. The research included comprehensive characterization of nanoparticles, in vitro studies of nanoparticles’ interactions with cells (mouse microvascular endothelial cells (bEnd.3) and astrocytes from brain rat cortex, and in vivo studies using the mouse model. It should be noted that nanoparticles used in animal studies did not contain any special tags facilitating their crossing through the blood-brain barrier. Any traces of PVA, which were weakly adsorbed onto the particles, were removed by the repeated sequence of the centrifugation-isolation-resuspension steps. The most important results of the aforementioned studies are as follows: The orally administered memantine-loaded nanoparticles with diameters lower than 200 nm were transported from the gastrointestinal tract to the blood. Circulating with blood, they crossed the blood-brain barrier and, according to histological analysis, accumulated in brain tissue. The behavioral tests revealed that animals treated with the memantine-loaded nanoparticles had better learning capability than mice treated with unencapsulated memantine.

Recently, Q. Wu et al. reported interesting studies on treating the primary mouse cortical cultured neurons with PLGA nanoparticles (632 nm diameter) [188]. The suspension of nanoparticles in the phosphate-buffered saline (0.01 M, pH 7.4) was prepared by dispersing the commercial product of Phosphorex (Hopkinton, MA, USA). For details of experiments, see Ref. [188]. The most important findings revealed that uptake of pure PLGA by neurons not only protects them against amyloid-*β* (A*β*) toxicity but reduces the conversion of the amyloid-β-protein precursor (APP) to A*β*. However, the aforementioned studies are at an early stage, and the mechanism of PLGA action on neurons requires more research.

Nowadays, chemotherapy is a standard procedure consisting of using drugs that, selectively and irreversibly internalized in the cancer cells, cause cell death or stop their proliferation (cytotoxic and cytostatic effects, respectively). In the case of brain cancer, these requirements are especially important because problems with crossing the BBB barrier hinder the delivery of drugs to the brain, and insufficient selectivity may be detrimental to the neurons, resulting in serious side effects. Some examples of recent research based on using polyester nanoparticle carriers are listed below. All these carriers were made from PLGA and its derivatives; all were designed for the preparation of drugs against glioma, the most often encountered primary brain cancer; however, they differed in detail to test various administration strategies.

Recently, Ye et al. proposed a new strategy based on targeting the epidermal growth factor receptors (EGFRs) [189]. These receptors are overexpressed on glioma cells, and their mutations and amplification result in the growth and proliferation of gliomas. Growth of glioma is also facilitated by Golgi phosphoprotein 3 (GPH3). Since Gefitinib (an anti-tyrosine kinase inhibitor) hampers EGFR production and GOLPH3 siRNA blocks the production of GPH3, authors prepared the angiopep-2 (A2)-modified cationic lipidpoly (lactic-co-glycolic acid) (PLGA) nanoparticles loaded with the aforementioned active substances. The in vitro and in vivo studies revealed the anti-glioma effects, justifying further studies.

In recent years, researchers turned their attention to hybrid nanoparticles of biological-synthetic origin with the hope of better tailoring their blood-brain barrier (BBB) and blood-brain tumor barrier (BBTB). As an example, may be mentioned PLGA nanoparticles coated with erythrocyte membrane and modified with two peptides from bacteria, known to enhance transport across BBB [190]. The synthesized nanoparticles loaded with Euphorbia factor L1 (EFL1) extracted from Euphorbia semen (model drug) were able to cross the BBB and BBTB and kill the glioma cells.

It is worth mentioning that still there is some space for the preparation of new drugs using the more traditional formulation methods, such as the preparation of PLGA/PLGA-PEO nanoparticles loaded with paclitaxel and R-flurbiprofen [191]. The nanoparticles were prepared by the nanoprecipitation method and by coating them with cationogenic chitosan. The amphiphilic PLGA-PEO copolymer was used to maintain the colloidal stability of nanoparticle suspension and chitosan coating to facilitate their uptake across the negatively charged membrane of glioma cells. The nanoparticles were administered by intraperitoneal route to rats with implanted tumor cells. It was noticed that animals treated with nanoparticles loaded with paclitaxel and R-flurbiprofen exerted anti-tumor activity.

Improvement in selectivity in targeting glioma cells required also basic research. Therefore, it is worth mentioning the work by S. Acharya et al. who investigated the effect of the folic acid moieties introduced into the interfacial layer of the prednisolone-loaded PLGA nanoparticles [192]. The studies proved that the presence of the folic acid labels strongly enhances the uptake of the nanoparticles by the cancer cells and that the anti-tumor activity of the nanoparticles is long-lasting.

The last example in this subsection describes the preparation and efficacy of the temozolomide-loaded PLGA nanoparticles, which were coated with fused stroma and glioma cell membrane fragments [193]. The nanoparticles were designed to target the glioma tumor microenvironment (TME), a structure containing glioma and stroma cells. Determination of the efficacy of the said nanoparticles as an anti-tumor agent was the main objective of the study. The drug-loaded PLGA nanoparticles were prepared by the oil-in-water emulsification solvent evaporation method. The membranes were obtained by lysis of the glioma and stroma cells, purification of the cell fragments, and their fusion. The nanoparticle coating was performed by several times repeated extrusion of the nanoparticles/membranes suspension through the 200 nm Whatman membrane filter. The details of preparation are described in Ref. [193]. It has been found that the treatment of mice with implanted gliomas using temozolomide-loaded PLGA nanoparticles targeting the gliomas TME extended animals’ lives.

## 5. Preclinical and Clinical Studies of Polyester Nanocarriers

Hundreds of research papers have been published on model drug polyester carrier systems. Far fewer are reports of preclinical studies using cell cultures, animals, or both. Recently, H.O. Alsaab et al. prepared a list of selected examples of preclinical studies based on nanocarriers made of PLGA polymers, or copolymers containing PLGA blocks [194]. These preclinical studies were related to the treatment of the following health problems (in parentheses are indicated the encapsulated drugs): lung cancer (platinum-curcumin or sorafenib), colon cancer (afatinib or 5-fluorouracil-chrisin), ovarian cancer (curcumin), and prostate cancer (uncaria tomentosa extract). The aforementioned publication (Ref. [184]) provides also information on the clinical trials of some PLGA-based formulations (data from the clinicaltrials.gov website of the US National Institute of Health). The following list contains names of the drugs and, in parentheses, indications for applications of drugs: Arestin (periodontal disease), Atridox^®^ (chronic adult periodontitis), Eligard (advanced prostatic cancer), Decapepty (prostate cancer), Lupron Depot (prostate cancer), Nutropin Depot^®^ (growth deficiency), Pamorelin^®^ (prostate cancer), Risperidal^®^ Consta (antipsychotic), Sandostatin^®^ LAR (acromegaly and carcinoid), Somatuline^®^ LA (acromegaly), Suprecur^®^ MP (prostate cancer), TrelstarTM Depot (advanced prostatic cancer), Zoladex^®^ (breast cancer and prostate cancer).

Four years ago, M. do Carmo Pereira published an interesting editorial paper discussing problems on the way from in vitro through preclinical and clinical studies to the pharmaceutical market [195]. Discussed were the immune nanocarriers for the targeted brain delivery. These nanocarriers are decorated with covalently immobilized antibodies specific to the receptors present mainly on the cancer cells. The most significant progress based on the aforementioned strategy was made for the liposomes and solid lipid nanoparticles. Some formulations of this kind (produced by Gilead Sciences, Foster City, CA, USA, and Cephalon/TEVA Pharmaceutical Industries, Fairfield, NJ, USA) are already on the market. Available are also some formulations based on the PLGA copolymers: Nutropin Depot^®^ (Genentech, South San Francisco, CA, USA) and Trelstar^®^ (Pfizer, New York, NY, USA) [195]. M. do Carmo Pereira et al. discussed also the strategy utilizing the receptor-ligand-mediated transport of nanoparticles across the blood-brain barrier. The aforementioned approach included the identification of receptors highly expressed on the surface endothelial cells of the capillary blood vessels, preparation of monoclonal antibodies against these receptors, and covalent binding of the antibodies to the surface of the drug-loaded nanoparticles. Such nanoparticles bind to receptors on the surface of endothelium cells of capillary blood vessels of the brain and enter into them by endocytosis, initiating the process of transport across the blood-brain barrier. Among the receptors are not only receptors for the most abundant transferrin and insulin but also receptors of folic acid, lipoprortein, lactoferrin, and some others. However, despite positive results in the in vitro studies, none of them was yet approved for use in clinics [195]. More successful were attempts at the development of polyester nanocarriers targeting other organs than the brain. Samyang Biopharmaceuticals (Seongnam-si, Republic of Korea) elaborated on the PEO-PLA polymeric micelle paclitaxel-loaded formulation. In 2007, this formulation under the name Genexol-PM was approved in South Korea for clinical application for the treatment of breast, lung, and ovarian cancers (information from the US National Institute of Health website updated in 2023 [196]. In 2018, S.-W. Lee et al. published an open-access article describing the results of the phase II trial to evaluate the efficacy and safety of a Cremophor-free polymeric micelle formulation of paclitaxel as a first-line treatment for ovarian cancer [197]. However, until 2023, no information is available about approval by the US Food and Drug Administration (FDA) or European Medicines Agency (EMA) for using Genexol-PM for anticancer therapy.

Since Genexol-PM micelles are stabilized with copolymers containing PEO blocks, it is worth mentioning a very important problem, which should attract the attention of the scientific community involved in studies in the field of drug delivery. The problem is related to the immunogenic properties of poly(ethylene oxide) (PEO), known also under the name poly(ethylene glycol) (PEG). In 2007, the paper entitled “PEGylated liposomes elicit an anti-PEG IgM response in a T cell-independent manner” [198] was published. The studies described in this publication proved that the intravenous injection of PEGylated liposomes, which did not contain any protein or peptide, results in weak IgG and intensive IgM responses. Later studies revealed that siRNA lipoplexes with surfaces containing bonded PEG are quickly cleared from the circulating blood when they are injected again after the first injection [199]. Further studies showed the that injection of siRNA-containing PEGylated lipoplex in mice results in PEG interaction with B cell-intrinsic toll-like receptor 7, leading to an immune response manifested by enhanced production of anti-PEG IgM [200]. As a result of the aforementioned research, it became clear that PEO should not be used for nanoparticle coating enhancing their circulation in blood but should be replaced by another non-immunogenic polymer. Probably, the immunogenicity of PEO/PEG developed with time due to the long-lasting exposure of humans to these water-soluble synthetic polymers commonly used in pharmacy, cosmetics, and even as food additives. However, the replacement of PEO or PEG with another synthetic polymer may be only a temporary solution because the problem may repeat.

## 6. Conclusions

A review of previous and recent publications discussing the synthesis of aliphatic polyesters of carboxylic acids shows that to date the researchers have at their disposal a rich library of homo- and copolymers with basic properties suggesting their suitability for using them for the preparation of drug nanocarriers. The simplest are the semi-crystalline poly(*ε*-caprolactone) (PCL) and highly crystalline polyglycolide (PGL). Another source-based name for PGL is poly(glycolic acid) (PGA), depending on whether the polymer is made by ring-opening polymerization from glycolide (the cyclic dimer of glycolic acid) or by polycondensation of the glycolic acid. Both PCL and PGA have only the methylene groups in the aliphatic partis, separating the ester groups. However, their thermal properties, solubility in organic solvents, and degradability differ significantly due to the very different microstructure. In PCL, the flexible aliphatic segment is long, consisting of five methylene groups. In PGA, only one methylene group separates two consecutive ester groups, making the PGA chains more rigid. As a result, for PCL Tg and melting temperatures are low, −60 and 60 °C, respectively, whereas for PGA Tg is in a range of 35 to 40 °C, and melting temperature ranges from 200 to 225 °C. Moreover, PCL is easily soluble in chloroform, dichloromethane, dimethyl sulfide, acetyl chloride, tetrahydrofuran, and furan that can be used in the processes of nanocarriers’ preparation [201], whereas PGA is soluble only in hexafluoroisopropanol, which makes the preparation of nanocarriers from PGA homopolymers very difficult. The problem was solved by replacing PGA with copolymers of glycolic acid and lactic acid (PLGA). Thus, most of the research on polyester drug carriers has been completed for polylactide/polyglycolide nano- and microparticles. It should also be stressed that the final formulation of the nanocarriers is free from the residual traces of the organic solvents. The current state of knowledge makes it possible to synthesize polylactides with the required stereoregularity and controlled crystallinity. It is possible to synthesize polylactides with the required molar masses in the range of a few thousand to about 6 × 10^5^ g/mol and with a narrow molar mass dispersion. Proper selection of methods used for the preparation of drug carriers allows for obtaining them with diameters from a few dozen nanometers to microns. The time of the drug release from polylactide and poly(lactide/glycolide) particles can be controlled to some extent from days to weeks. A significant advance in the synthesis of polylactides is the development of its metal-free synthesis. The process yields polymers that do not contain heavy metals and, at least by some researchers, are considered safer for medical applications. Noteworthy is the polymerization initiated by systems with initiator and catalytic groups combined into a single molecule, so that they later remain permanently bound to the polymer chain and thus cannot migrate outside the drug carrier particle.

A very special case is poly(*β* butyrolactone) (PBL) and poly(hydroxybutyrate) (PHB) (two source names are used for a polymer with the same chain structure, depending on whether the polymer was derived from *β*-butyrolactone or hydroxybutyric acid). Their synthesis and biosynthesis have been comprehensively studied, and their overall structure-property relationships suggest their usefulness for medical applications, but what is surprising is that research in this area is still scarce and the field requires more extensive exploration. Since PLAs and PBLs contain chiral carbon atoms for the preparation of nanocarrier polymers with well-defined content of the stereoisomeric units and with the known microstructure, they should be used. There is great potential in using macrolactones with double bonds to synthesize functional nanoparticles for targeted drug delivery. However, so far no reports on the preparation of such nanoparticles are available. However, there are articles on the copolymerization of ε-caprolactone and macrolactones with double bonds and their subsequent functionalization in the click thiol-ene reaction. The relationship between drug release and nanoparticle degradation is not well understood. The need for multiple-drug-loaded nanoparticles and sequential (or triggered by external stimuli) drug release from them is well recognized. It should be noted that there were obtained PLGA nanocarriers that, even without any targeting moieties, can efficiently cross the blood-brain barrier, opening the way for the simple synthesis of drugs for the patients suffering from Alzheimer’s and Parkinson’s diseases. Thus, there is still a lot of space for further research in the field of polyesters tailored for drug carriers.
